# Ultra‐Thin Oxide‐Based Double‐Layer Architecture Achieves Wide‐Temperature Broadband Microwave Absorption by Synergizing Lorentz Resonance and Thermionic Transport

**DOI:** 10.1002/advs.202515679

**Published:** 2025-11-03

**Authors:** Zewen Duan, Ruopeng Cui, Yi Li, Lu Gao, Xuefei Zhang, Lingfeng Yuan, Biao Zhao, Chunlei Wan

**Affiliations:** ^1^ Transportation Institute Inner Mongolia University Hohhot 010020 China; ^2^ State Key Laboratory of New Ceramics and Fine Processing, School of Materials Science and Engineering Tsinghua University Beijing 100084 China; ^3^ College of Mathematics and Physics Beijing University of Chemical Technology Beijing 100029 China; ^4^ Aviation Key Laboratory of Science and Technology on Advanced Surface Engineering AVIC Manufacturing Technology Institute Beijing 100024 China; ^5^ School of Microelectronics Fudan University Shanghai 200433 China

**Keywords:** lorentz dielectric resonance, macroscopic interfacial resonance, rare‐earth zirconates, thermionic transport, wide‐temperature adaptive microwave absorption

## Abstract

Achieving high and stable permittivity is essential for developing ultra‐thin and wide‐temperature adaptive microwave‐absorbing materials (MAMs). Conventional high‐permittivity MAMs suffer from temperature‐sensitive permittivity and cannot achieve stable microwave‐absorbing performance at fluctuating temperatures, while temperature‐insensitive MAMs exhibit low permittivity that severely restricts ultra‐thin designs. Herein, a double‐layer La_2_Zr_2_O_7_/Eu_2_Zr_2_O_7_ architecture is employed, with rare‐earth zirconates featuring with one‐eighth anion‐site vacancies, as a promising candidate for ultra‐thin wide‐temperature adaptive MAMs. The established balance between thermionic relaxation polarization and Lorentz dielectric resonance—activated by oxygen ions—can largely increase the real part of permittivity of La_2_Zr_2_O_7_ while maintaining its stability at different temperatures. Concurrently, strong thermionic transport induces an increase in dielectric loss capacity of Eu_2_Zr_2_O_7_, which can broaden effective absorption bandwidth (EAB) effectively. Moreover, the macroscopic interfacial resonance between two layers overcomes the limitations imposed by the quarter‐wavelength theory, generating new absorption peak for further expanding EAB. Under synergistic coupling of these mechanisms, it is successfully achieved superior EAB—completely covering the Ku band (12.4–18 GHz) over a wide‐temperature range (400–800 °C) at an ultra‐thin fixed thickness of just 1.2 mm, coupled with a maximum EAB/*d* of 3.6 GHz mm^−1^. This innovative design offers promising pathways for developing ultra‐thin and wide‐temperature adaptive MAMs.

## Graphical abstract

Developing ultrathin wide‐temperature adaptive microwave absorption materials (MAMs) is crucial for enhancing the survival of military devices under alternating high‐temperature environment. Here, a double‐layer La_2_Zr_2_O_7_/Eu_2_Zr_2_O_7_ architecture is employed, with rare‐earth zirconates featuring with one‐eighth anion‐site vacancies, successfully achieving broadband and stable microwave‐absorbing performance over a wide‐temperature range (400–800 °C) at an ultra‐thin fixed thickness of merely 1.2 mm. This innovative design offers a promising pathway for developing ultra‐thin and wide‐temperature adaptive MAMs.

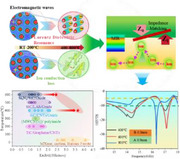

## 1 Introduction

The rapid advancement of information technology has made microwaves in the Ku band indispensable for modern applications, from wireless communications to radar systems, owing to their high data transmission rates.^[^ ^1^, ^2^, ^3^, ^4^ ^]^ This widespread use has simultaneously driven the need for high‐performance microwave‐absorbing materials (MAMs) capable of mitigating electromagnetic pollution in healthcare, electronic safety, and electromagnetic stealth for national defense security such as aerospace vehicles.^[^ ^1^, ^2^, ^3^, ^4^, ^5^, ^6^, ^8^ ^]^ Particularly, with the increasing agility of aerospace vehicles and the variation of flight speeds (subsonic or supersonic),^[^ ^9^, ^10^ ^]^ more and more devices in high‐speed aerospace vehicles, e.g., nose cone and aero‐engines, will be confronted with the fluctuant high‐temperature environments alongside radar detection.^[^ ^11^, ^12^, ^13^ ^]^ These conditions require MAMs to maintain stable microwave‐absorbing performance across wide‐temperature ranges at a specific fixed thickness without compromising their stealth efficiency.

To enable stable microwave absorption across wide‐temperature ranges, MAMs should generally satisfy two critical requirements simultaneously: (1) exceptional resistance to high‐temperature oxidation, as well as (2) stable permittivity with appropriate dielectric loss capacity. Conventional MAMs can be categorized into two main types: magnetic‐loss materials (e.g., Fe/Co/Ni alloys^[^ ^14^, ^15^, ^16^ ^]^) and dielectric‐loss materials (notably 2D‐based materials^[^ ^17^, ^18^ ^]^ and carbon‐based composites^[^ ^19^, ^20^, ^21^ ^]^). Although these MAMs demonstrate outstanding room‐temperature microwave‐absorbing performance due to their tunable permittivity and diverse loss mechanisms, they encounter significant limitations in high‐temperature applications. Magnetic materials suffer from severe performance degradation above their Curie points due to the loss of ferromagnetism,^[^ ^22^, ^23^ ^]^ while 2D‐based or carbon‐based MAMs usually become susceptible to oxidation when exposed to temperatures above 400 °C.^[^ ^24^, ^25^ ^]^ These intrinsic limitations result in a substantial deterioration in the electromagnetic parameters of these MAMs, fundamentally constraining their applications in high‐temperature microwave‐absorbing scenarios.

In comparison, some ceramic‐based materials—with robust oxidation resistance and minimal permittivity fluctuations across wide‐temperature ranges—are seemingly considered as ideal candidates for wide‐temperature adaptive MAMs in alternating high‐temperature conditions. Extensive research has focused on non‐oxide SiC‐based and Si_3_N_4_‐based materials, such as SiC_f_/SiC‐SiC_nw_,^[24]^ SiC/SiO_2_,^[^ ^26^, ^27^ ^]^ CNTs/SiO_2_,^[28]^ SiC_f_/Si_3_N_4_,^[^ ^29^, ^30^ ^]^ SiC_nw_/C/Si_3_N_4_,^[31]^ SiC/Si_3_N_4,_^[32]^ and TiC_x_N_1‐x_ fibers/Si_3_N_4._^[33]^ These materials can exhibit stable microwave‐absorbing performance over wide‐temperature ranges at a specific fixed thickness owing to their minimal permittivity fluctuations and suitable dielectric loss capacity at different temperatures. However, these MAMs share a common drawback: the optimal microwave‐absorbing performance can be only achieved at relatively large thicknesses (*d* > 3 mm),^[^ ^24^, ^26^, ^27^, ^29^, ^30^, ^31^, ^32^, ^33^ ^]^ which are contrary to the desired ultra‐thin design necessary for the lightweight developing trend. This serious drawback stems from their inherently low permittivity, primarily due to the insufficient degree of dielectric loss mechanisms at GHz frequencies.

Based on the Maxwell's equations, high permittivity is an essential factor for minimizing the thickness of MAMs.^[^ ^34^, ^35^ ^]^ To enhance the permittivity as well as dielectric loss of ceramic‐based MAMs, researchers have explored a series of targeted strategies.^[^ ^7^ ^]^ The primary approaches involve introducing controllable conduction (e.g., electron transport) or polarization mechanisms (e.g., dipole and interfacial polarization) into the ceramic matrix, which enables flexible regulation of dielectric properties over a wide temperature range, as well as the synergistic optimization of impedance matching and attenuation capability. In terms of the electrical conduction mechanisms, they have attempted to incorporate conductive ceramics with high oxidation resistance, such as Ti_3_SiC_2_,^[^ ^36^, ^37^, ^38^ ^]^ TiC,^[^ ^39^ ^]^ TiC_x_N_1‐x_ fiber,^[^ ^33^ ^]^ La_0.9_Sr_0.1_MnO_3_^[25]^ and MgZr_4_P_6_O_24._^[40]^ With regard to polarization mechanism, they have employed doping modification technique or lattice distortion/defects engineering strategy (vacancies, interstitial and heterointerface etc.) to facilitate effective dipolar or interfacial polarization within these materials, including Ni‐decorated SiC,^[^ ^41^ ^]^ TiN/ZrO_2_/C,^[^ ^42^ ^]^ and sandwich‐like SiCnw/C/Si_3_N_4._^[31]^ These MAMs can exhibit high permittivity and diversified dielectric loss mechanisms, contributing to the optimized high‐temperature microwave‐absorbing performance, i.e., broad effective absorption bandwidth (EAB > 3 GHz) and satisfying minimum reflection loss (*R*_Lmin_←40 dB) within relatively thinner thicknesses (*d* ≤ 2.5 mm).^[^ ^38^, ^41^, ^43^ ^]^ Nevertheless, the permittivity of these MAMs exhibits pronounced temperature dependence, undergoing significant fluctuations with different temperatures.^[^ ^28^, ^43^, ^44^, ^45^, ^46^ ^]^ In these cases, achieving optimal microwave‐absorbing performance across a wide‐temperature range necessitates frequently adjusting the thickness of MAMs to compensate for the permittivity fluctuations with different temperatures, which poses a great obstacle to realize stable microwave absorption across a wide‐temperature range at a fixed thickness. Therefore, the primary challenge in designing ultra‐thin MAMs for wide‐temperature adaptive stealth is the intractable conflict between ultra‐thin design and stable microwave absorption. The key to solving this issue lies in balancing two competing demands: (1) maximizing permittivity for thin designs as much as possible, while (2) minimizing its thermal sensitivity for stable microwave absorption. In other words, it still remains a great challenge to achieve stable microwave‐absorbing performance (*R*_L_ ≤ −5 dB) in a wide‐temperature range under a fixed thickness of less than 1.5 mm because of their fundamental incompatibility between the ultra‐thin designs and stable permittivity at different temperatures.^[^ ^24^, ^25^, ^26^, ^27^, ^28^, ^29^, ^30^, ^31^, ^36^, ^37^, ^38^, ^39^, ^40^, ^41^, ^42^, ^43^, ^44^ ^]^

In this work, we introduce a double‐layer structural design comprising rare‐earth zirconate ceramics (REZCs), employing upper La_2_Zr_2_O_7_ and lower Eu_2_Zr_2_O_7_ layers with one‐eighth anion site vacancies. This design endows the La_2_Zr_2_O_7_/Eu_2_Zr_2_O_7_ double‐layer structure with dual advantages of high permittivity and thermal stability—a critical breakthrough that successfully overcomes the incompatibility between the ultra‐thin designs and stable permittivity at different temperatures, realizing the integration of ultra‐thin and wide‐temperature adaptive microwave absorption for the first time. Specifically, the oxygen anions in La_2_Zr_2_O_7_ and Eu_2_Zr_2_O_7_ induce distinct dielectric loss mechanisms. The robust Lorentz dielectric resonance excited by oxygen anions in La_2_Zr_2_O_7_ can amplify the real part of the permittivity while maintaining its stability under fluctuant temperature. By employing La_2_Zr_2_O_7_ as the upper layer for impedance matching, the overall stability of microwave absorption regarding the double‐layer structure is significantly improved. In contrast, the increase in dielectric loss capacity triggered by the strengthened thermionic transport at elevated temperatures in the lower Eu_2_Zr_2_O_7_ lossy layer substantially broadens the EAB of the double‐layer structure. Moreover, the macroscopic interfacial resonance occurred between the La_2_Zr_2_O_7_ and Eu_2_Zr_2_O_7_ layers overcome the limitations imposed by the quarter‐wavelength theory, generating new absorption peak for further expanding the EAB given the fixed thickness. Under the coupling effect of these diversified dielectric loss mechanisms, the double‐layer La_2_Zr_2_O_7_/Eu_2_Zr_2_O_7_ structure obtains superior EAB, almost completely covering the Ku band (12.4–18 GHz) over a wide‐temperature range (400–800 °C), at an ultra‐thin and fixed thickness of merely 1.2 mm. Notably, it maintains a maximum EAB/*d* of 3.6 GHz/mm at 400–800 °C, coupled with a maximum |*R*_Lmin_/*d*| of 31.3 dB mm^−1^ at 800 °C—far outperforming that of the reported high‐temperature MAMs. This innovative design of anion‐sites‐assisted double‐layer rare‐earth zirconate with multiscale structural coupling provide an effective pathway for the development of advanced ultra‐thin and wide‐temperature adaptive MAMs.

## 2 Results

### 2.1 Microstructure Characterizations and Temperature‐Dependent Dielectric Properties of Rare‐Earth Zirconates

**Figure 1:**
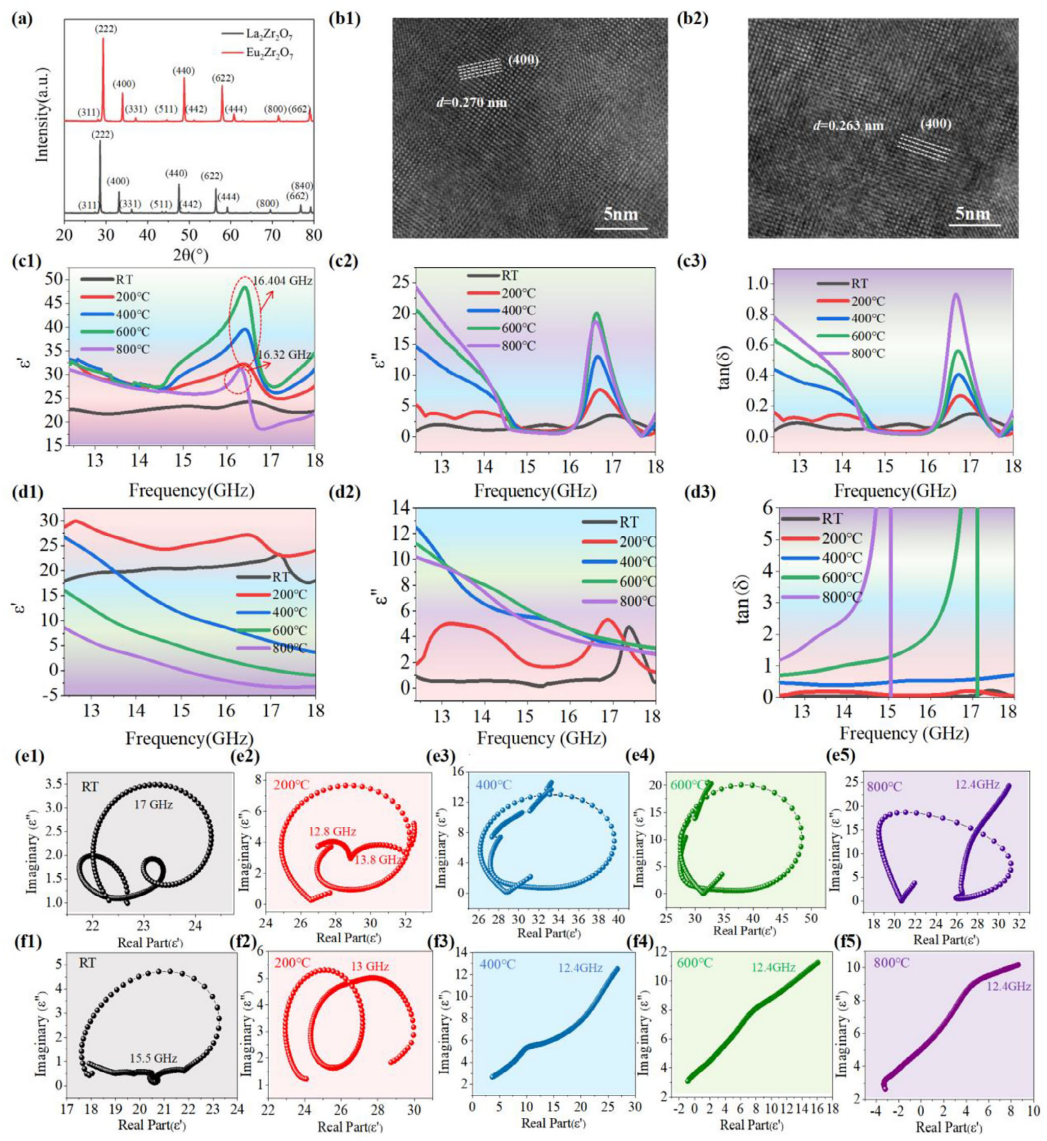
Lattice characterization, phase composition, and temperature‐dependent dielectric properties of Ln_2_Zr_2_O_7_ (Ln: La, Eu). a) XRD patterns of La_2_Zr_2_O_7_ and Eu_2_Zr_2_O_7_. b1–2) HRTEM images of La_2_Zr_2_O_7_ and Eu_2_Zr_2_O_7_ sintered at 1600 °C. c1–3) *ε*′, *ε*“ and tan*δ* of La_2_Zr_2_O_7_. d1–3) *ε*′, *ε*” and tan*δ* of Eu_2_Zr_2_O_7_. e1–5) Cole‐Cole diagrams of La_2_Zr_2_O_7_. f1–5) Cole‐Cole diagrams of Eu_2_Zr_2_O_7_.

As shown in **Figure** **1**a, the as‐synthesized Ln_2_Zr_2_O_7_ (Ln = La, Eu) samples exhibit nearly similar X‐ray diffraction pattern. The diffraction peaks at (311), (331), and (511) corresponding to the characteristic peaks of the pyrochlore structure are evident in the XRD patterns, which demonstrates the successful formation of La_2_Zr_2_O_7_ and Eu_2_Zr_2_O_7_ phases.^[^ ^47^, ^48^ ^]^ The nanoscale microstructure of these zirconates is further characterized by the high‐resolution transmission electron microscope (HRTEM). Ion‐thinning samples for HRTEM observation and EDS elemental mapping images are presented in Figures S1–S3 (Supporting Information). As shown in Figure 1b1,2, the atoms of La_2_Zr_2_O_7_ and Eu_2_Zr_2_O_7_ are neatly arranged. The inverse Fast Fourier transform (iFFT) was performed to clearly reveal the lattice features of La_2_Zr_2_O_7_ and Eu_2_Zr_2_O_7_. The results indicate that although La_2_Zr_2_O_7_ and Eu_2_Zr_2_O_7_ possess one‐eighth anion‐site vacancies, there are nearly no lattice distortion and imperfections in these zirconates, as presented in Figure S4 (Supporting Information). A comparable observation has been made in Gd_2_Zr_2_O_7._^[49]^ This can be attributed to the symmetry of the overall lattice and the interactions between ions, the ordered arrangement of rare‐earth ions and Zr^4+^, and the charge compensation mechanisms, which collectively inhibit the formation of lattice distortions. A typical SEM micrograph of these zirconates is presented in Figure S5 (Supporting Information). The crystal grains in micron‐size are general equiaxed in shape, and there is no evidence of a second phase or noticeable pores, suggesting the high densification of the sintered La_2_Zr_2_O_7_ and Eu_2_Zr_2_O_7_ ceramic bulks, which was consistent with the prior literature.^[^ ^50^ ^]^

**Figure 2:**
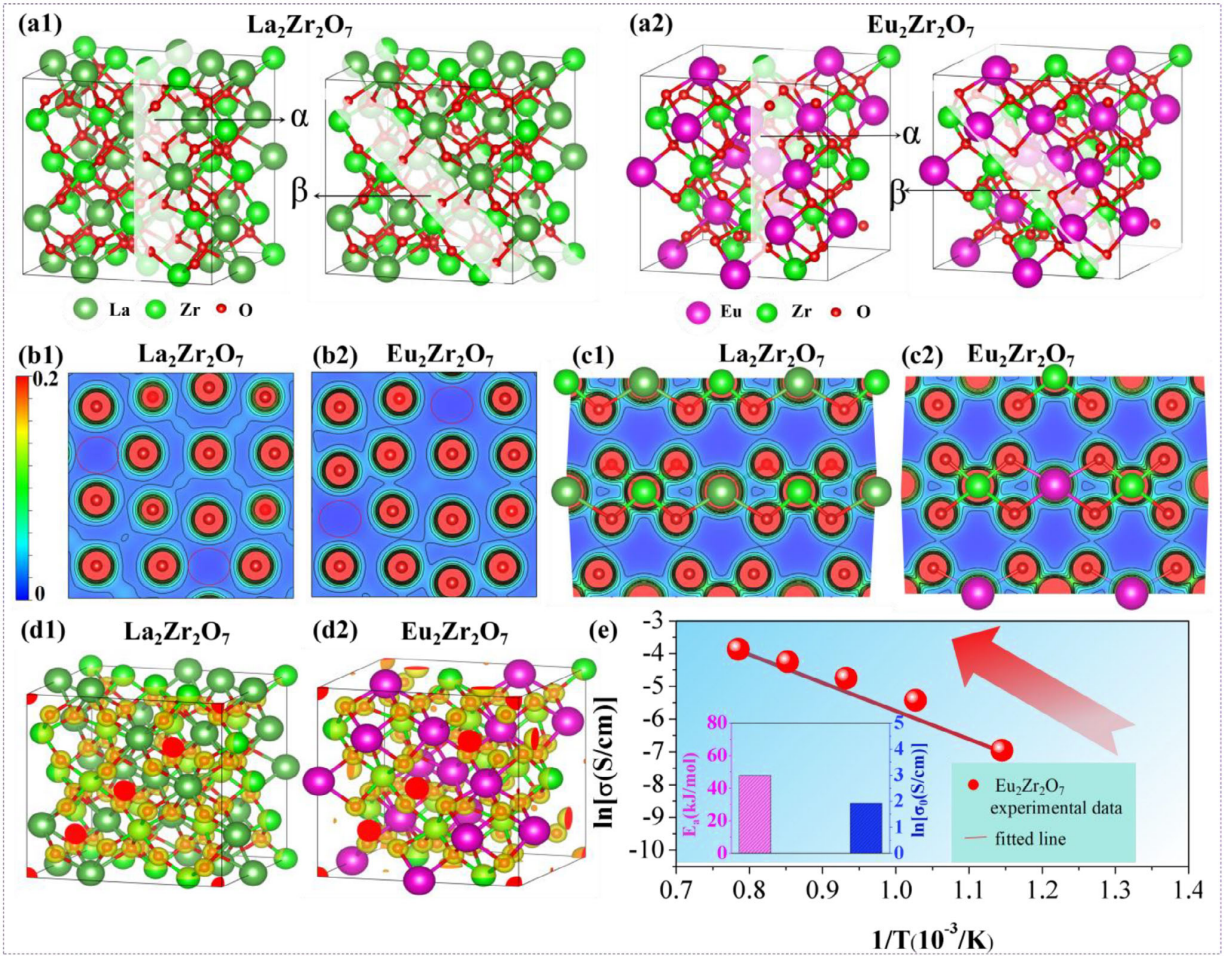
Ln_2_Zr_2_O_7_ (Ln: La, Eu) lattice structure model for first principles calculations and dielectric loss mechanisms. a1,2) Selected different crystal planes (α–β) in the lattices of La_2_Zr_2_O_7_ and Eu_2_Zr_2_O_7_ for charge density calculations. b1,2) 2D charge density maps of crystal planes α. c1,2) 2D charge density maps of crystal planes β. d1,2) 3D charge density maps of La_2_Zr_2_O_7_ and Eu_2_Zr_2_O_7_. e) Ionic conductivity of Eu_2_Zr_2_O_7_ as well as fitted lines, with inset of preexponential factor and apparent activation energy from the Arrhenius equation.

In general, the relative complex permittivity (*ε*_r_ = *ε*'‐j*ε*“) was critical parameters for determining the dielectric properties and microwave absorption of REZCs. The real part of permittivity (*ε*') represents the storage capability of microwave energy, while the imaginary part of permittivity (*ε*”) denotes the ability of microwave energy dissipation.^[^ ^14^, ^15^, ^17^, ^19^, ^20^, ^21^ ^]^ Figure 1c1–3, d1–3 illustrates the frequency‐dependent and temperature‐dependent *ε*', *ε*“ and tan*δ* of La_2_Zr_2_O_7_ and Eu_2_Zr_2_O_7_ in the Ku band (25–800 °C). At room temperature (25 °C), both La_2_Zr_2_O_7_ and Eu_2_Zr_2_O_7_ displays an anomalous increase in *ε*' with frequency (Figure 1c1,d1). Nevertheless, an intriguing phenomenon emerges as the temperature further rises to 200–800 °C, where La_2_Zr_2_O_7_ and Eu_2_Zr_2_O_7_ demonstrate distinct permittivity characteristics. As observed in Figure 1c1,2, within the frequency range of 12.4‐14.5 GHz, both the *ε*' and *ε*” of La_2_Zr_2_O_7_ exhibit a decreasing trend with frequency from 200 to 800 °C, which indicates the presence of a dielectric relaxation mechanism in La_2_Zr_2_O_7_ that is driven by thermionic relaxation polarization with short‐range oxide‐ion transport. Notably, the *ε*' curves display a maximum and minimum just below and above specific frequencies, respectively, whereas *ε*″ curves and attenuation constant (*α*, as shown in Figure S6, Supporting Information) display distinct sharp peaks in the 16–17.5 GHz range, along with subtle fluctuations in the 14.5–16 GHz range. These characteristics provide an indicator of Lorentz dielectric resonance.^[^ ^51^, ^52^, ^53^ ^]^ As the temperature rises, the intensity of Lorentz dielectric peaks significantly enhances. Meanwhile, with the frequency range of 12.4–14.5 GHz, the *ε*' demonstrates a high degree of overlap across temperatures ranging from 200 to 800 °C, manifesting that it remains essentially unchanged with temperature. Furthermore, as the temperature rises to 800 °C, the frequency of Lorentz dielectric resonance peak undergoes a slight decrease compared to the 16.404 GHz observed in the 200–600 °C, eventually dropping to 16.32 GHz. The validity of this dielectric resonance can be further corroborated by the Cole‐Cole plots of La_2_Zr_2_O_7_. As demonstrated in Figure 1e2–5, inverted semicircles or even complete circles appear, deviating from the conventional upright semicircle shape that manifests the traditional Debye dielectric relaxation model.^[^ ^8^, ^54^ ^]^ Therefore, the aforementioned analysis indicates that dielectric polarization behaviors of La_2_Zr_2_O_7_ exhibits both Debye relaxation and Lorentz dielectric resonance. Previous researches have indicated that such dielectric resonance in some ceramics may be closely associated with intrinsic point defects within the lattice.^[^ ^55^, ^56^ ^]^ As demonstrated in **Figure** **2**b1,2, the charge density maps elucidate the inherent oxygen vacancies defects (highlighted in red circle) within the crystal unit cells of La_2_Zr_2_O_7_ and Eu_2_Zr_2_O_7_, revealing the high concentrations of intrinsic oxygen vacancies in REZCs. These vacancies primarily originate from the non‐equivalence in theoretical valence states between Ln atoms and Zr atoms in REZCs, and ideally, they should be electrical neutrality. Additionally, based on the EPMA measurement, the O/Zr atomic ratio in La_2_Zr_2_O_7_ and Eu_2_Zr_2_O_7_ is less than 3.5 (Figure S7, Supporting Information), implying that apart from the intrinsic oxygen vacancies, there are extra positively charged oxygen vacancies in REZCs to ensure overall electrical neutrality. These extra oxygen vacancies may share some positive charges with the inherent ones, which effectively stabilizes the lattice structure of REZCs. Concurrently, oxygen anions in REZCs serve as negatively charged counterparts to the oxygen vacancies, establishing electric dipoles that are capable of triggering Lorentz dielectric resonance under the alternating electromagnetic field. This can be vividly demonstrated by the charge density contrast in Figure 2b1,2, where oxygen atoms in these REZCs are highlighted in deep red, signifying electron accumulation, while oxygen vacancies are displayed in deep blue, indicating electron depletion. Furthermore, as evidenced in Figure 2b1,2,c1,2, the ordered pyrochlore‐phase La_2_Zr_2_O_7_ and Eu_2_Zr_2_O_7_ exhibit similar electron delocalization phenomena. To balance internal energy and stabilize the lattice structure, La_2_Zr_2_O_7_ and Eu_2_Zr_2_O_7_ only require moderate electron delocalization within the unit cell due to the ordered distribution of oxygen vacancies in pyrochlore‐phase REZCs unit cells.

In contrast to La_2_Zr_2_O_7_, Eu_2_Zr_2_O_7_ demonstrates remarkably distinct complex permittivity characteristics. Within the 25–200 °C range, Eu_2_Zr_2_O_7_ displays only a weak Lorentz dielectric resonance, located within the 16–17.5 GHz (Figure 1d1). As the temperature rises to 400–800 °C, this dielectric resonance vanishes completely. Simultaneously, the *ε*' of Eu_2_Zr_2_O_7_ shows significant fluctuations with changes in temperature, with no curve overlap across the entire Ku‐band. Concurrently, *ε*' of Eu_2_Zr_2_O_7_ undergoes a sharp frequency‐dependent decline, accompanied by pronounced dispersion effects. As the temperature rises to 600 and 800 °C, *ε*' continues to decline and goes below zero, exhibiting Lorentz‐type negative permittivity behavior around 18 GHz. Such Lorentz‐type negative permittivity responses are also observed in the frequency range of 10 MHz to 1 GHz.^[^ ^57^, ^58^, ^59^ ^]^ This phenomenon is not attributed to internal polarization mechanisms,^[^ ^60^, ^61^ ^]^ but is closely associated with the conductive phase.^[^ ^60^, ^61^, ^62^ ^]^ These results indicate that Lorentz‐type negative permittivity of Eu_2_Zr_2_O_7_ will be closely correlated to their conductivity at elevated temperature. Prior literature has reported that rare‐earth zirconates generally display a characteristic of oxide‐ion conduction at elevated temperatures, a behavior primarily enabled by the long‐range migration of oxygen ions through lattice vacancies.^[^ ^63^, ^64^ ^]^ Herein, the temperature dependence of conductivity of Eu_2_Zr_2_O_7_ was characterized based on Arrhenius Equation.^[^ ^65^ ^]^ As shown in Figure 2e, the Arrhenius plots (1/*T*, ln*σ*) of Eu_2_Zr_2_O_7_ nearly aligned with a straight line, which indicated the enhanced ionic conduction with increasing temperature.^[^ ^66^ ^]^ When the temperature was raised to 800 °C, ion conductivity was up to 8.64 × 10^−3 ^S cm^−1^, demonstrating a substantial increase of several orders of magnitude compared with that at 400 °C. Additionally, its larger *ε*″ observed at 400–800 °C compared to that at 25–200 °C (Figure 1d2), coupled with their straight linear tail of Cole‐Cole plot at 400–800 °C (Figure 1f3–5), further corroborates the enhanced ionic conductivity of Eu_2_Zr_2_O_7_ at high temperatures. Although the *ε*″ exhibits minimal variation across the 400–800 °C range (Figure 1d2), the *α* demonstrates a pronounced increase with temperature, which correlates well with the rise in ionic conductivity. As shown in Figure S8 (Supporting Information), *α* exhibits a positive correlation with conduction loss—in agreement with previous reports,^[^ ^34^, ^67^, ^68^ ^]^ which unequivocally underscores the significant contribution of ionic conduction to dielectric loss of Eu_2_Zr_2_O_7_. At elevated temperatures, the long‐range migration of oxygen ions, which leads to a high ion conductivity, inevitably disrupts the electric dipoles equilibrium induced by oxygen anions and vacancies, driving the transition from Lorentz dielectric resonance primarily induced by oxygen vacancy defects to Lorentz‐type ionic conduction behavior, as schematically depicted in Figure 5c. Moreover, within the 400–800 °C range, *ε*″ demonstrates a gradual frequency‐dependent decline in the Ku band, in contrast to the steep drop of *ε*'. This disparity in *ε*' and *ε*″ attenuation rates leads to a progressive increase in tan*δ* with frequency, as depicted in Figure 1d3 and Figure S9 (Supporting Information). According to quarter‐wavelength theory,^[^ ^69^ ^]^ such a frequency‐dependent increase in tan*δ* is advantageous for broadening EAB.

Compared with Eu_2_Zr_2_O_7_, La_2_Zr_2_O_7_ exhibits significantly lower conductivity, reaching only ≈0.2 × 10^−3 ^S·cm^−1^ even at 800 °C.^[^ ^66^ ^]^ This low conductivity issues from a distinct conduction mechanism compared to other REZCs. Hiroshi et al.^[^ ^66^ ^]^ investigated the correlation between the conductivity of Ln_2_Zr_2_O_7_ (Ln = La, Er, Eu, Gd and Sm) and oxygen partial pressure *p*(O_2_), revealing that while most compositions (Ln≠La) exhibit oxide‐ion conduction as the dominant mechanism, La_2_Zr_2_O_7_ displays unique behavior—its conductivity positively correlated with *p*(O_2_), characteristic of hole conduction. Consequently, the contribution of oxygen atoms to ionic migration in La_2_Zr_2_O_7_ is negligible within the temperature of RT‐800 °C, and the long‐range migration of oxygen atoms is highly restricted, both of which guarantee the stability of the number of electric dipoles that are essential for sustaining strong Lorentz dielectric resonance. As depicted in Figure 1c1, La_2_Zr_2_O_7_ maintains a prominent Lorentz dielectric resonance from 200 to 600 °C, with its intensity increasing due to enhanced dipole polarization at elevated temperatures. This mechanism was schematically illustrated in Figure 5c. Nevertheless, at 800 °C, the Lorentz dielectric resonance peak begins to decrease, primarily due to the growing degree of thermionic relaxation polarization. Unlike long‐range ion conduction mechanism, thermionic relaxation polarization involves short‐range migration of oxygen ions by abundant oxygen vacancies within the lattice, requiring lower activation energy due to the lower potential barriers for oxygen atoms to move over a short distance.^[^ ^70^ ^]^ Consequently, the onset of thermionic relaxation polarization inevitably disrupts the equilibrium of electric dipoles, particularly at 800 °C, partially attenuating the Lorentz dielectric resonance. This effect constrains the resonance peak within a finite and reasonable range, suppressing excessive temperature‐dependent amplification and thereby maintaining system stability. This established balance between Debye thermionic relaxation polarization and Lorentz dielectric resonance—activated by oxygen ions—can largely increase the *ε*' of La_2_Zr_2_O_7_ while maintaining its stability at different temperatures.

The microwave‐absorbing performance of these zirconates can be evaluated by reflection loss (*R*_L_) and effective absorption bandwidth (EAB).^[^ ^71^ ^]^ The *R*_L_ values with normal incidence of microwaves were calculated by using the widely accepted single‐layer transmission line theory with a metal backing.^[^ ^72^ ^]^ As presented in Figures S10 and S11 (Supporting Information), although the single‐layer La_2_Zr_2_O_7_ within stable *ε*' exhibits relatively poor microwave absorption performance, it can maintain stable *R*_L_ curves across various temperature ranges (400–800 °C) at the same thickness. In contrast, single‐layer Eu_2_Zr_2_O_7_ with unstable *ε*' exhibits a larger fluctuation in *R*_L_ within temperature ranges of 400–800 °C at the same thickness, but it displays a relatively broader EAB and a higher |*R*_L_| (Figures S12 and S13, Supporting Information). The 3D *R*_L_ plots of La_2_Zr_2_O_7_ and Eu_2_Zr_2_O_7_ are presented in Figures S14 and S15 (Supporting Information). In summary, La_2_Zr_2_O_7_ always exhibits pronounced Lorentz dielectric resonance effects and maintains stable *ε*' within the 12.4–14.5 GHz across temperatures from 200 to 800 °C. This stability enables our proposed double‐layer structure to retain stable microwave absorption over a wide temperature range with a fixed thickness. Conversely, Eu_2_Zr_2_O_7_ exhibits weak Lorentz dielectric resonance at lower temperatures (25–200 °C). As temperatures exceed 400 °C, there is a transition from Lorentz dielectric resonance toward ionic conduction, causing frequency‐dependent rise in tan*δ*, which is advantageous for expanding EAB.

### 2.2 Ultra‐Thin and Wide‐Temperature (400–800 °C) Adaptive Microwave Absorption of Double‐layer La_2_Zr_2_O_7_/Eu_2_Zr_2_O_7_ Architecture

Although single‐layer La_2_Zr_2_O_7_ exhibits stable *R*_L_ curves within 400–800 °C at a fixed thickness, its absolute *R*_L_ value (|*R*_L_|) remains relatively low. Constrained by the quarter‐wavelength theory of single‐layer MAMs, its EAB fails to effectively cover the Ku band. In the case of single‐layer Eu_2_Zr_2_O_7_, the EAB broadens as the temperature increases from 25 to 600 °C. Nevertheless, as temperature rises to 800 °C, its high ionic conductivity intensifies the primary reflection of electromagnetic waves (EMW), leading to a significant reduction in the EAB, as illustrated in Figures S12c–e (Supporting Information). Consequently, achieving a wide EAB and stable absorption performance over a wide temperature range, while maintaining a fixed ultra‐thin thickness, poses a formidable challenge for single‐layer rare‐earth zirconates. To overcome this limitation, a double‐layer La_2_Zr_2_O_7_/Eu_2_Zr_2_O_7_ architecture has been designed, which satisfies the fundamental requirements of being “thin, wide, and strong” for high‐performance microwave‐absorbing materials while largely mitigating temperature‐dependent absorption instability. As previously discussed, La_2_Zr_2_O_7_ exhibits substantial Lorentz dielectric resonance and obtains stable *ε*′ values between 200–800 °C, making it an ideal upper matching layer to optimize impedance matching over a wide temperature range. In contrast, Eu_2_Zr_2_O_7_ undergoes a transition from Lorentz dielectric resonance to ionic conduction at elevated temperatures. Its strong ionic conduction loss and the unique characteristic of frequency‐dependent increase in tan*δ* render it a suitable lower lossy layer for broadening the EAB. Using the multilayer transmission line theory with a metal backplate,^[^ ^73^ ^]^ the normalized input impedance (*Z*_in_) of the double‐layer La_2_Zr_2_O_7_/Eu_2_Zr_2_O_7_ structure relative to free space can be derived to evaluate its microwave‐absorbing performance.

**Figure 3:**
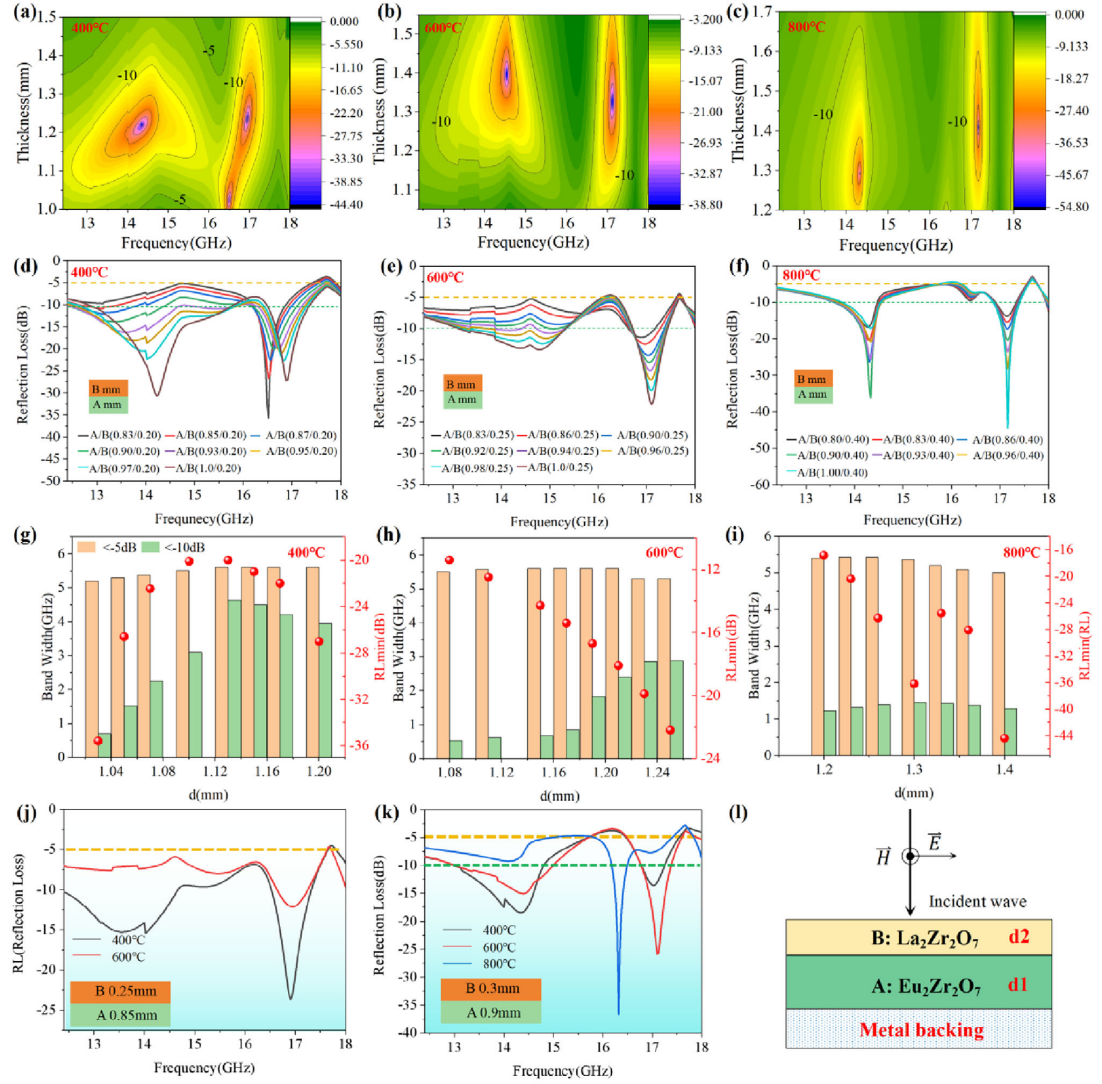
Double‐layer microwave‐absorbing performance of rare‐earth zirconates. a–c) 2D color‐mapping of reflection loss for double‐layer La_2_Zr_2_O_7_/Eu_2_Zr_2_O_7_ architecture at 400, 600, and 800 °C with the fixed thickness of 0.2, 0.25, and 0.4 mm for La_2_Zr_2_O_7_ layer. d–f) Frequency‐dependent *R*_L_ curves of double‐layer La_2_Zr_2_O_7_/Eu_2_Zr_2_O_7_. g–i) EAB and *R*_Lmin_ of double‐layer with different thickness from 400 to 800 °C. j) Frequency‐dependent *R*_L_ curves of double‐layer La_2_Zr_2_O_7_/Eu_2_Zr_2_O_7_ with the same thickness of 1.1 mm at temperature of 400–600 °C. k) Frequency‐dependent *R*_L_ curves of La_2_Zr_2_O_7_/Eu_2_Zr_2_O_7_ with same thickness of 1.2 mm at temperature of 400–800 °C. l) Schematic illustration of three‐layer square model with the incidence of TM waves utilized for RCS simulation.

**Figure 4:**
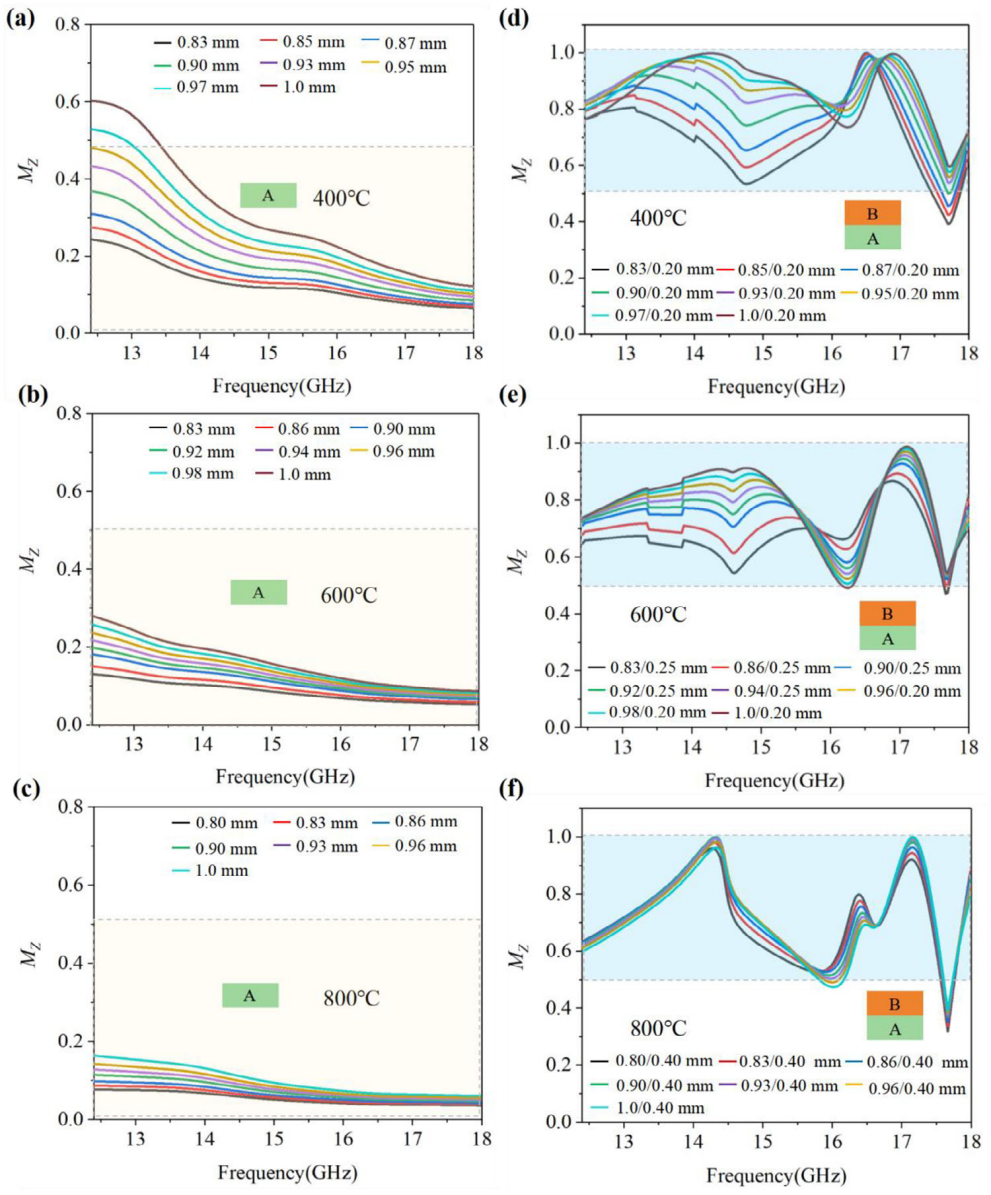
Impedance matching of double‐layer La_2_Zr_2_O_7_/Eu_2_Zr_2_O_7_ architecture. a–c) The frequency dependence of *M*z curves of single‐layer Eu_2_Zr_2_O_7_. d–f) The frequency dependence of *M*z curves of double‐layer La_2_Zr_2_O_7_/Eu_2_Zr_2_O_7_ structure.

**Figure 5:**
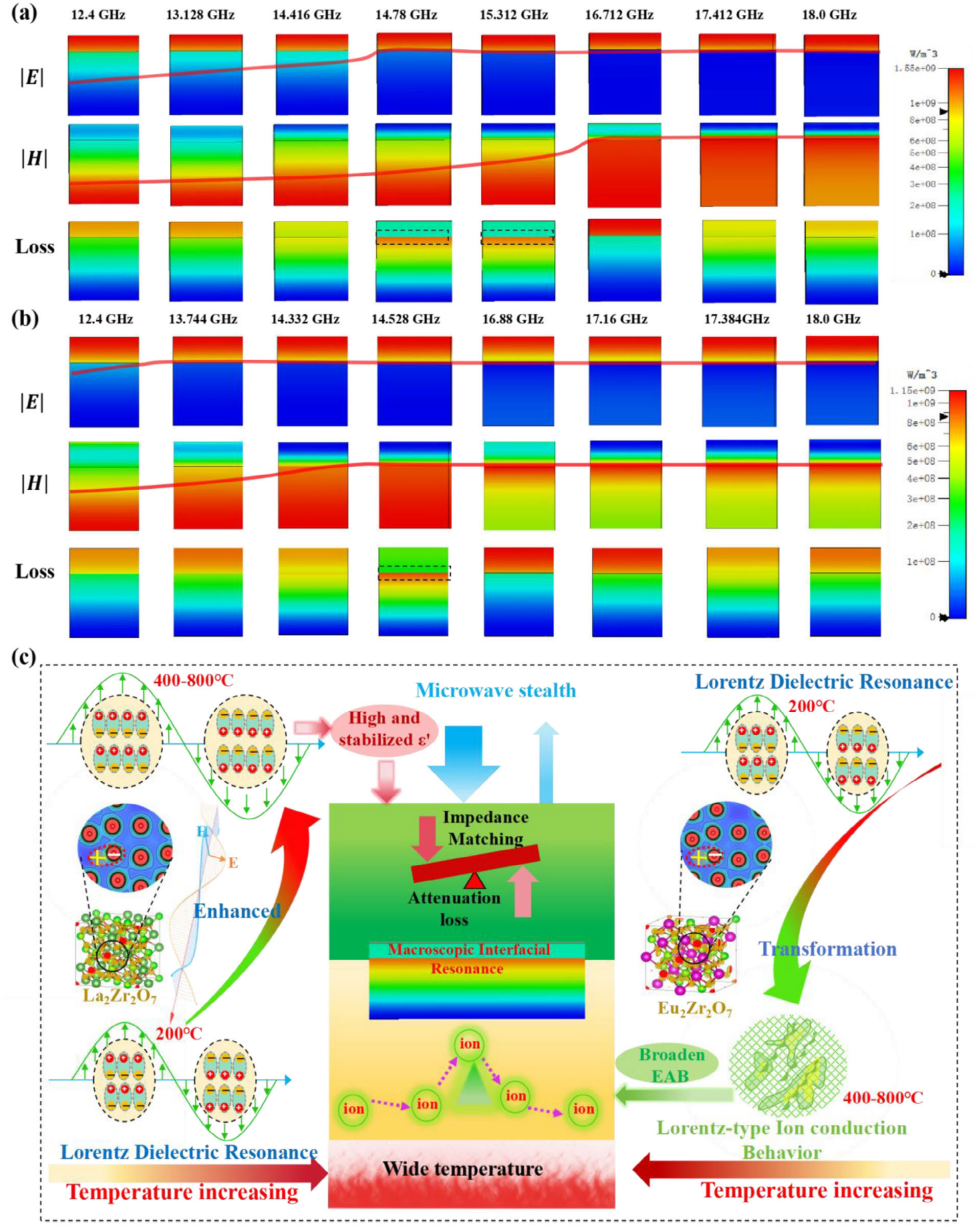
Analysis of the microwave‐absorbing mechanisms of the double‐layer La_2_Zr_2_O_7_/Eu_2_Zr_2_O_7_ structure. a) Simulations of *E*‐field, *H*‐field, and energy loss distributions of the double‐layer structure with thicknesses of 0.25 mm/1 mm at 600 °C monitored at different frequencies. b) Simulations of *E*‐field, *H*‐field, and energy loss distributions of the double‐layer structure with thicknesses of 0.4 mm/1 mm at 800 °C monitored at different frequencies. c) Schematic illustration of the various synergistic microscopic‐macroscopic mechanisms for the double‐layer La_2_Zr_2_O_7_/Eu_2_Zr_2_O_7_ architecture.

**Figure 6:**
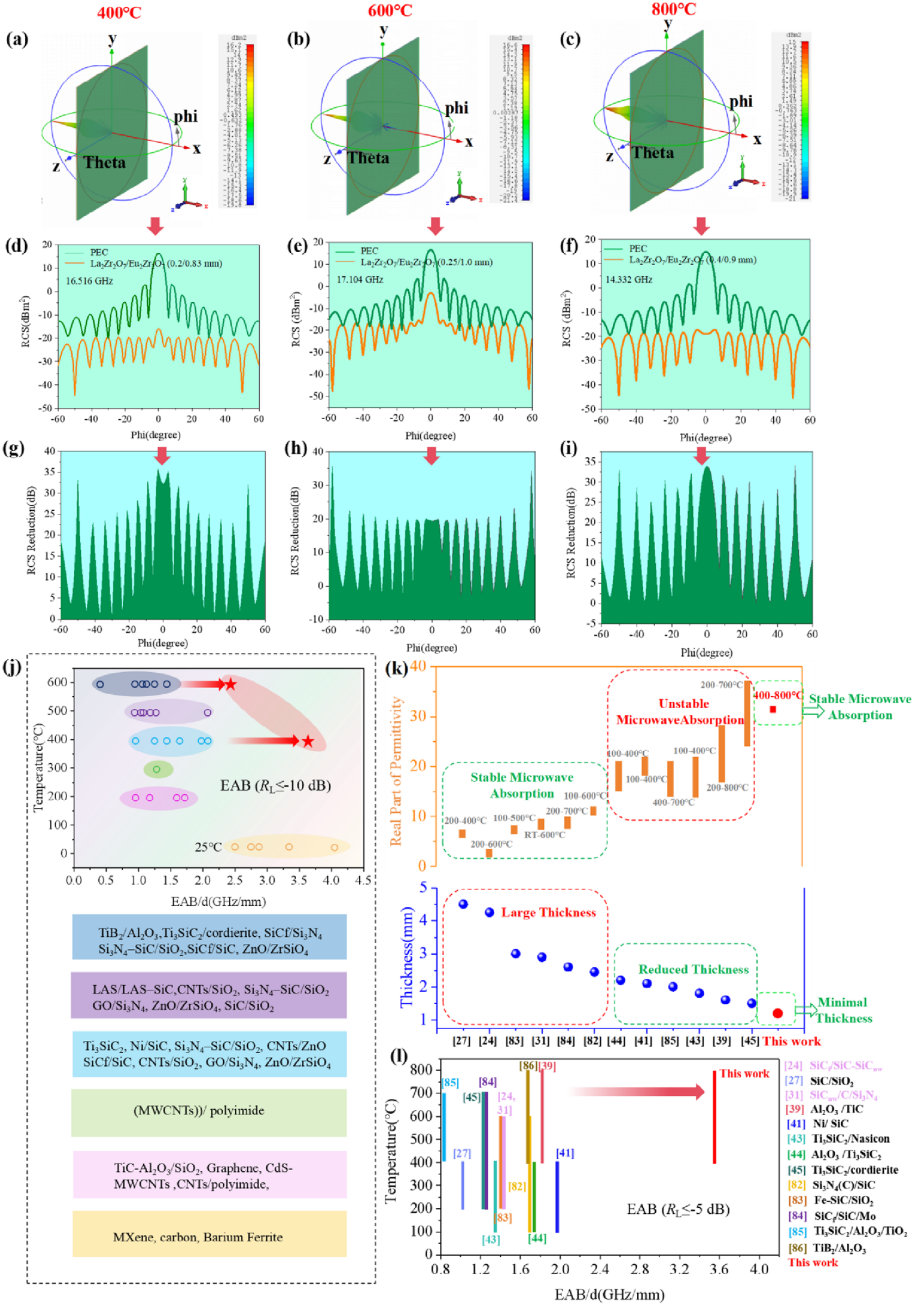
Far‐field RCS simulations of double‐layer La_2_Zr_2_O_7_/Eu_2_Zr_2_O_7_ at 400, 600, and 800 °C. a) 3D RCS image of the double‐layer structure at 400 °C (0.2 mm/0.83 mm, at 16.516 GHz). b) 3D RCS image of the double‐layer structure at 600 °C (0.25 mm/1.0 mm, at 17.104 GHz). c) 3D RCS image of the double‐layer structure at 800 °C (of 0.4 mm/0.9 mm, at 14.332 GHz). d‐f) RCS curves of the double‐layer structure at 400, 600, and 800 °C. g–i) RCS reduction curves of the double‐layer structure at 400, 600, and 800 °C. j) Comparison of microwave‐absorbing performance (*R*_L_ ≤ −10 dB) of this work and previously reported MAMs at different high temperature. k) A comparative analysis temperature‐induced permittivity variations and their correlation with absorber thickness. l) Comparison of wide‐temperature adaptive microwave‐absorbing performance at the corresponding thicknesses with other high‐temperature MAMs see refs. [82‐86].

**Figure** **3** shows the 2D color‐mapping of *R*_L_ curves, EAB and *R*_Lmin_ of the double‐layer La_2_Zr_2_O_7_/Eu_2_Zr_2_O_7_, where the thickness of the impedance matching layer (La_2_Zr_2_O_7_) was fixed while the thickness of the lossy layer (Eu_2_Zr_2_O_7_) was systematically regulated. Remarkably, compared to the microwave‐absorbing performance of single‐layer La_2_Zr_2_O_7_ or Eu_2_Zr_2_O_7_ (Figure S16, Supporting Information), the double‐layer La_2_Zr_2_O_7_/Eu_2_Zr_2_O_7_ structure demonstrates wider EAB and enhanced |*R*_Lmin_| values across 400–800 °C. Specially, at 400 °C, this double‐layer structure achieves a maximum EAB/*d* value of 4.07 GHz/mm (*R*_L_≤‐10 dB) at 1.13 mm, with a *R*_Lmin_ of ‐37 dB (Figure 3d). When the temperature was evaluated to 600 °C, the value of EAB/*d* reaches 2.3 GHz mm^−1^ at 1.23 mm (*R*_L_≤‐10 dB), accompanied by a *R*_Lmin_ of −23 dB (Figure 3e), which significantly outperform the previously reported high‐temperature MAMs (Figure 6j). Further heating to 800 °C yields an EAB/*d* of 1.1 GHz mm^−1^ at 1.3 mm (*R*_L_ ≤ −10 dB), coupled with a *R*_Lmin_ of ‐44 dB (Figure 3f) and a maximum |*R*_Lmin_/*d*| of 31.3 dB mm^−1^. More importantly, with the reference standard of *R*_L_ ≤ −5 dB, this double‐layer structure presents superior EAB, nearly covering the Ku band across 400–800 °C at an ultra‐thin and fixed total thickness of just 1.2 mm (Figure 3k), which successfully realizes the integration of ultra‐thin and wide‐temperature adaptive microwave absorption for the first time (Figure 6k). Notably, it consistently sustains a remarkable maximum EAB/*d* of 3.6 GHz mm^−1^ (0.3 mm/0.9 mm) over 400–800 °C, far surpassing the previously reported wide‐temperature adaptive MAMs (Figure 6l). Interestingly, the *R*_L_ curve fluctuations of single‐layer La_2_Zr_2_O_7_ (Figures S16a–c, Supporting Information) and the double‐layer structure (Figure 3k) exhibit striking similarity, which strongly underscores that while the lower lossy layer (Eu_2_Zr_2_O_7_) is highly sensitive to temperature variations, the upper matching layer (La_2_Zr_2_O_7_)—with its temperature‐insensitive *ε*′—plays a dominant role in stabilizing the overall microwave absorption of double‐layer structure. Beyond thermal stability, the enhanced microwave‐absorbing performance and broadened EAB of this double‐layer structure primarily emanate from two synergistic mechanisms: (1) optimized impedance matching (**Figure** **4**), and (2) amplified power loss driven by the macroscopic interface resonance between the La_2_Zr_2_O_7_ and Eu_2_Zr_2_O_7_ layers, as revealed by CST simulations in **Figure** **5**a,b.

Compared with the single‐layer La_2_Zr_2_O_7_ with only one absorption peak arising from the Lorentz dielectric resonance at 16.5 GHz (Figure S16a–c, Supporting Information), the double‐layer La_2_Zr_2_O_7_/ Eu_2_Zr_2_O_7_ structure display two prominent absorption peaks (*R*_Lmin_←10 dB) within the Ku band, as demonstrated in Figure 3d–f. The newly emerged absorption peak at 14–14.5 GHz in the double‐layer structure can be ascribed to the hierarchical interference cancellation effect triggered by the opposing distributions of electric and magnetic fields in each layer.^[^ ^74^ ^]^ As shown in Figure 5a,b, the interface of La_2_Zr_2_O_7_/Eu_2_Zr_2_O_7_ exhibits the enhanced power loss intensity. Specifically, power loss was observed at 14.78 and 15.312 GHz for double‐layer structure with thickness of 0.25 mm/1.0 mm at 600 °C, and at 14.528 GHz for structure with thickness of 0.4 mm/1.0 mm at 800 °C. These power losses occur at frequencies near the new absorption peak identified in Figure 3e,f. This underscores the crucial role of macroscopic interface resonance in creating new absorption peaks in the La_2_Zr_2_O_7_/Eu_2_Zr_2_O_7_ double‐layer structure. Different from the intrinsic dielectric loss mechanisms of these REZCs, this newly emerged macroscopic interface resonance introduced an additional structural‐response‐based loss mechanism, further enhancing electromagnetic dissipation and broadening the EAB while reducing the *R*_Lmin_. This helps to overcome the constrains imposed by the quarter‐wavelength theory, thereby improving the impedance matching coefficient (*M*_Z_) of the double‐layer structure. The *M*_Z_, characterizing the degree of impedance matching, is presented in Figure 4. As the *M*_Z_ is equal to 1, perfect impedance matching will be achieved,^[^ ^75^ ^]^ manifesting optimal MA performance. As depicted in Figure 4a–c, the *M*_Z_ of single‐layer Eu_2_Zr_2_O_7_ at a fixed thickness tends to decrease as the temperature rises. This decline is primarily due to a substantial increase in ionic conductivity that leads to stronger initial reflection of EMW from the material surface. Although the *M*_z_ of Eu_2_Zr_2_O_7_ rises with increasing thickness, it reaches only a maximum of 0.6 at 1 mm—still far below the ideal value of 1. By contrast, the design of a double‐layer structure with La_2_Zr_2_O_7_ as the upper layer over Eu_2_Zr_2_O_7_ significantly enhanced the overall impedance matching. The *M*_Z_ curves of this double‐layer are closer to 1 at multiple frequency points (Figure 1d–f), revealing that more EMW can enter into the MAMs and be dissipated via thermionic conduction. This analysis demonstrates that the double‐layer structure design significantly enhances the electromagnetic impedance matching performance. Beyond double‐layer design, approaches such as multilayer designs, metamaterial incorporation, and device integration also offer promising avenues for further balancing *M*_Z_ and attenuation constant.^[^ ^76^, ^77^, ^78^, ^79^, ^80^, ^81^ ^]^

### 2.3 Microwave Stealth Ability of the Double‐Layer La2Zr2O7/Eu2Zr2O7 Architecture at 400–800 °C

To access the microwave stealth performance of the designed double‐layer architecture, the radar cross section (RCS) of the double‐layer La_2_Zr_2_O_7_/Eu_2_Zr_2_O_7_ structure at different temperature was simulated under far‐field conditions by using CST software. The monitoring frequencies of 16.516, 17.104, and 14.332 GHz were selected due to their corresponding minimum reflection loss of in the double‐layer structure with thicknesses of ≤1.3 mm. RCS is defined as the effective area of an object that reflects the same reflected radar echo signals as a metal sphere with equivalent projected area, where lower RCS values indicate better stealth performance. **Figure** **6**a–c exhibits the 3D RCS simulation images of the double‐layer structure at different temperature under various detecting angles. The simulated plates coated with the MAMs displays lower scattering signals than original PEC plates at 400 and 800 °C, where the RCS curve of the double‐layer La_2_Zr_2_O_7_/Eu_2_Zr_2_O_7_ nearly drops below −20 dBm^[^ ^2^ ^]^ across the angle range of [−60°, 60°] (Figure 6d,f), comparable to the RCS of birds, which confirms that these designed MAMs exhibit effective microwave stealth applicability. Notably, the RCS reduction value of the double‐layer La_2_Zr_2_O_7_/Eu_2_Zr_2_O_7_ structure can exceed 32 dB at 400 or 800 °C with a detection angle of 0° (Figure 6g,i), exceeding the majority of reported ceramic‐based MAMs. The 3D RCS simulation of double‐layer structure (0.2 mm/1.0 mm) at the absorption frequency of 14.22 GHz (400 °C) was presented in Figure S17 (Supporting Information). This remarkable performance underscores the potential of the La_2_Zr_2_O_7_/Eu_2_Zr_2_O_7_ double‐layer design for stealth applications.

## 3 Conclusion

In summary, we proposed a double‐layer La_2_Zr_2_O_7_/Eu_2_Zr_2_O_7_ architecture featuring one‐eighth anion‐site vacancies and remarkable thermal stability, enabling ultra‐thin and wide‐temperature adaptive microwave absorption. The results demonstrate that oxygen vacancies in REZCs trigger diversified electromagnetic loss mechanisms. Specially, the abundant oxygen vacancies in La_2_Zr_2_O_7_ induce strong Lorentz dielectric resonance at elevated temperatures, which greatly amplifies its real part of the permittivity while maintaining its stability under fluctuant temperature. By employing La_2_Zr_2_O_7_ as the upper layer for impedance matching, the overall microwave absorption stability of the double‐layer structure is thereby significantly enhanced. Meanwhile, the oxygen vacancies in lower Eu_2_Zr_2_O_7_ lossy layer lead to the transition from Lorentz dielectric resonance to thermionic conduction. The frequency‐dependent increase in tan*δ* triggered by thermionic transport substantially broadens the effective absorption bandwidth of the double‐layer structure. Moreover, the intense macroscopic interfacial resonance between the La_2_Zr_2_O_7_ and Eu_2_Zr_2_O_7_ layers overcomes the limitations imposed by the quarter‐wavelength theory, forming new absorption peak for further expanding the EAB. Under the coupling effect of these various electromagnetic loss mechanisms, this La_2_Zr_2_O_7_/Eu_2_Zr_2_O_7_ double‐layer structure can basically cover the Ku band over a broad temperature range (400–800 °C) at a fixed ultra‐low thickness of 1.2 mm. This study provides a novel insight for the development of ultra‐thin and wide‐temperature adaptive MAMs, which exhibits broad application prospects in the aerospace field.

## 4 Experimental Section

### Chemicals and Materials

High‐purity metal oxide powders, including La_2_O_3_ (purity ≥99.99%), Eu_2_O_3_ (purity ≥99.99%) and ZrO_2_ (purity ≥99.99%) were purchased from Aladdin Industrial Corporation (Shanghai, China). Ethanol (≥99.7%) was obtained by MREDA Technology Corporation (Beijing, China). All chemicals were used as received without any purification.

### Sample Preparation

Rare‐earth zirconates (La_2_Zr_2_O_7_ and Eu_2_Zr_2_O_7_) were synthesized via the solid reaction with raw powders of La_2_O_3_, Eu_2_O_3_, and ZrO_2_. Initially, these metal oxide powders were subjected to pre‐calcined at 1000 °C for 2 h to eliminate adsorbed CO_2_ and H_2_O. Subsequently, these powders were homogenously mixed in the stoichiometric ratio by ball milling (250 rpm, 5 h) in ethanol using ZrO_2_ balls as the grinding medium. The obtained slurries were subsequently dried by rotary evaporation and then sintered at 1250 °C for 5 h. Afterwards, the pre‐sintered powders were subjected to a second round of ball milling and rotary evaporation under the identical conditions to ensure homogeneity. After drying, the powders were thoroughly ground and sieved through a 200‐mesh screen to obtain fine particles for pressing. Disk samples were synthesized by hydraulic pressing at 20 MPa for 5 min to obtain green bodies, followed by cold isostatic pressing at 220 MPa. Finally, these samples underwent pressure less sintering in a furnace at 1600 °C for 10 h, and then cooled to room temperature to obtain the dense ceramic bulks.

### Structural and Compositional Characterizations

The phase characteristics of La_2_Zr_2_O_7_ and Eu_2_Zr_2_O_7_ were determined by X‐ray diffractometer (XRD, Rigaku D/max 2500) with Cu K*α* radiation (40 kV, 150 mA). Diffractograms were captured over a 2*θ* range of 20–80° at a scanning rate of 8°min^−1^. Microstructural morphology was examined using a scanning electron microscope (SEM, Zeiss Merlin, 15 kV). To enhance grain boudaries contrast in SEM observation, samples were subjected to thermal etching 1550 °C for 30 min, and subsequently coated with conductive platinum prior to SEM observation. Nanoscale microstructure was investigated via the high‐resolution transmission electron microscope (HRTEM, JEOL JEM‐2100F), with samples prepared by ion‐beam thinning. Elemental composition was verified using an electron probe microanalyzer (EPMA, JEOL JXA8230). Prior to EPMA measurement, all samples were polished and coated with conductive carbon to ensure a smooth and conductive surface.

## Conflict of Interest

C.W., R.C., and Z.D. have applied for a Chinese patent (CN118598661A) that has been published and is under substantive examination. The patent covers the synthesis and application of double‐layer thermal insulating microwave absorbers with rare‐earth zirconates mentioned in this work. The other authors declare no conflict of interest.

## Supporting information



Supporting Information

## Data Availability

The data that support the findings of this study are available from the corresponding author upon reasonable request.
