# Supplementary material for: Ultra‐Thin Oxide‐Based Double‐Layer Architecture Achieves Wide‐Temperature Broadband Microwave Absorption by Synergizing Lorentz Resonance and Thermionic Transport
Source: Adv Sci (Weinh). 2025 Nov 3;13(2):e15679. doi: 10.1002/advs.202515679 (PMC12786290; doi:10.1002/advs.202515679)
Supplement: Supplementary file 1 — Supporting Information [file ADVS-13-e15679-s001.docx]

**Supporting Information for**

**Ultra-Thin Oxide-based Double-layer Architecture Achieves Wide-Temperature Broadband Microwave Absorption by Synergizing Lorentz Resonance and Thermionic Transport**

Zewen Duan^1, 2†^, Ruopeng Cui^2, †^,Yi Li^4, †^, Lu Gao^5^, Xuefei Zhang^2^, Lingfeng Yuan^2^, Biao Zhao^3,^ *, Chunlei Wan^2,^ *

^1^Transportation Institute, Inner Mongolia University, Hohhot, 010020, China

^2^State Key Laboratory of New Ceramics and Fine Processing, School of Materials Science and Engineering, Tsinghua University, Beijing, 100084, China

^3^School of Microelectronics, Fudan University, Shanghai, 200433, China

^4^College of Mathematics and Physics, Beijing University of Chemical Technology, Beijing, 100029, China

^5^Aviation Key Laboratory of Science and Technology on Advanced Surface Engineering, AVIC Manufacturing Technology Institute, Beijing, 100024, China

^†^ These authors contributed equally to this work.

* Author to whom correspondence should be addressed.

E-mail: [zhao_biao@fudan.edu.cn](mailto:zhao_biao@fudan.edu.cn), [wancl@mail.tsinghua.edu.cn](mailto:wancl@mail.tsinghua.edu.cn)

Table of contents

**S1. Electrical and Electromagnetic properties measurement**

**S2. First principles calculations**

**S3. CST simulations**

**S1. Electrical and Electromagnetic properties measurement**

The ionic conductivity of Eu_2_Zr_2_O_7_ was evaluated using AC impedance spectroscpy (CHI660E impedance analyzer) across a temperature range of 600-1000 °C with a 100 °C increment. Measurement was performed under an applied AC signal with frequency sweeping from 100 Hz to 0.1 MHz. The temperature-dependent conductivity behavior follows the Arrhenius relationship^1, 2^:

*σ*=*σ_0_*exp(*-E*_a_/*RT*) (1)

where *σ* represents the ionic conductivity, the pre-exponential factor (*σ*₀) reflects the number of mobile ions, *E*ₐ is the apparent activation energy, *R* is the gas constant, and *T* is the temperature. As illustrated in Figure 2e, the natural logarithm of the ionic conductivity of Eu₂Zr₂O₇ remains extremely low (less than -10) at temperatures below 400 ℃, potentially introducing significant measurement uncertainties. Consequently, linear extrapolation proves more reliable than direct experimental measurement for determining the ionic conductivity of Eu₂Zr₂O₇ in the sub-600 °C regime. Additionally, the Lorentz dielectric resonance model can be expressed as follows^3-5^:

$\varepsilon^{'}=\varepsilon_{\infty}+\frac{\left( \varepsilon_{0}-\varepsilon_{\infty} \right)[1-{(f/f_{1})}^{2}]}{{[1-{(f/f_{1})}^{2}]}^{2}+{(f/f_{2})}^{2}}$ (2)

$\varepsilon^{''}=\frac{\left( \varepsilon_{0}-\varepsilon_{\infty} \right)(f/f_{2})}{{[1-{(f/f_{1})}^{2}]}^{2}+{(f/f_{2})}^{2}}$ (3)

where *ε*_∞_ denotes the optical permittivity, *ε*₀ corresponds to the static permittivity, and *f*_1_, *f*_2_ are characteristic frequency parameters.

To evaluate the microwave-absorbing performance, rectangular samples (15.80 mm×7.90 mm×2 mm) were cut from sintered ceramic bulks for Ku-band measurement. Using waveguide method, the complex permittivity of the samples was measured over the 12.4–18 GHz frequency range at various temperatures (25 °C, 200 °C, 400 °C, 600 °C, and 800 °C) using a vector network analyzer (VNA, Agilent E5071C) equipped with a heating device. During the measurement, the complex permeability was fixed at 1-j0 (*i.e.* non-magnetic mode). The essence of the “breakpoint” is actually due to the significant inconsistency in the variation of the *ε*' or *ε*" of the permittivity with the same frequency interval. The obtained electromagnetic parameters were used to calculate the *R*_L_-*f* curves according to the transmission line theory (Equation 4 and 5)^6, 7^:

$Z_{\mathrm{in}}=\sqrt{1/\varepsilon_{r}}\tanh(j\frac{2\pi fd}{c}\sqrt{\varepsilon_{r}})$ (4)

$R_{L}=20\log\left| \frac{Z_{\mathrm{in}}-1}{Z_{\mathrm{in}}+1} \right|$ (5)

where *Z*_in_ is the normalized input impedance of the single-layer rare-earth zirconates relative to the free space; j is the imaginary unit, *f* is the microwave frequency, *d* is the thickness of the single-layer rare-earth zirconates, c is the speed of light in a vacuum, and *ε*_r_=*ε*'-j*ε*'' is the relative complex permittivity of these zirconates. Attenuation Constant (*α*) of these samples was calculated by the following equation^8^:

*α*=$\frac{\sqrt{2}\pi f}{c}$×$\sqrt{(\varepsilon"\mu"-\varepsilon'\mu')+\sqrt{{(\varepsilon"\mu"-\varepsilon'\mu')}^{2}+{(\varepsilon"\mu"+\varepsilon'\mu')}^{2}}}$ (6)

To calculate the microwave-absorbing performance of the double-layer structure, the normalized input impedance (*Z*_in_) of the double-layer structure relative to free space can be derived via the multilayer transmission line theory with a metal backing^7^:

$Z_{in2}=\sqrt{1/\varepsilon_{2}}\tanh(j\frac{2\pi fd_{2}}{c}\sqrt{\varepsilon_{2}})$ (7)

$Z_{\mathrm{in}}=\sqrt{1/\varepsilon_{1}}\frac{Z_{in2}+\sqrt{1/\varepsilon_{1}}\tanh(j\frac{2\pi fd_{1}}{c}\sqrt{\varepsilon_{1}})}{\sqrt{1/\varepsilon_{1}}+Z_{in2}\tanh(j\frac{2\pi fd_{1}}{c}\sqrt{\varepsilon_{1}})}$ (8)

Where *Z*_in2_ denotes input impedance between the La_2_Zr_2_O_7_ and Eu₂Zr₂O₇ layers, and the thicknesses of the La_2_Zr_2_O_7_ and Eu_2_Zr_2_O_7_ layers are represented by *d*_1_ and *d*_2_, respectively. Additionally, *ε*_1_ and *ε*_2_ denote their relative complex permittivity of these zirconates. By substituting Equation (7) into Equation (5), the reflection loss of the double-layer La_2_Zr_2_O_7_/Eu_2_Zr_2_O_7_ structure can be accurately calculated. Furthermore, the *M*_z_ value calculated by Eqs. (9) is utilized to examine the critical factor for adjustable MA performance. Here, *Z'*_in_ denotes the real part of normalized input impedance.

$M_{z}=\frac{2{Z_{\mathrm{in}}}^{'}}{\left| Z_{\mathrm{in}} \right|^{2}+1}$ (9)

The quarter-wavelength matching model (*λ/4*) plays a great critical role in optimizing the practical application of single-layer MAMs, especially in realizing the interference- cancellation effect of the incident and reflected microwave, and it can be described as following equation^6, 8^:

*d*_m_=*n*$\frac{\lambda_{e}}{4}$*=n*$\frac{c}{4f\sqrt{\left| \varepsilon_{r} \right|\left| u_{r} \right|}}$ (n=1, 3, 5,…) (10)

**S2. First principles calculations**

Based on the density-functional theory (DFT), the electronic structures of La_2_Zr_2_O_7_ and Eu_2_Zr_2_O_7_ were calculated using the Vienna *ab* initio simulation package (VASP)^9^. The projector augmented wave (PAW) method was utilized to conduct the density functional calculations^10, 11^. The generalized gradient approximation (GGA) was employed as the electronic exchange correlation potential. The plane wave cutoff energy was established at 450 eV. For the unit cell of La_2_Zr_2_O_7_ and the 2×2×2 supercell of Eu_2_Zr_2_O_7_, a 3×3×3 Monkhorst-Pack k-point mesh was utilized to fully optimize the atomic positions. The convergence criteria for the total energy and the Hellmann-Feynman forces were set at 1×10^-7^meV and 0.5 meV/Å, respectively. Based on the optimized lattices of La_2_Zr_2_O_7_ and Eu_2_Zr_2_O_7_ lattices, the charge density was calculated.

**S3. CST simulations**

The microwave energy distribution (including *E*-field, *H*-field, and power loss density) and far-field bistatic radar cross section (RCS) of the double-layer La_2_Zr_2_O_7_/Eu_2_Zr_2_O_7_ structure were simulated using CST Studio Suite 2018. To analyze the distribution characteristics at elevated temperatures (400 °C, 600 °C, and 800 °C), three structural configurations with thicknesses of 0.2 mm/1.0 mm, 0.25 mm/1.0 mm and 0.40 mm/1.0 mm were modeled on the X-O-Y plane respectively. Corresponding cells were defined with these models, with periodic boundary conditions applied along the *x* and *y* directions. An electric boundary condition (*E*_t_=0) was imposed at the *z*_min_ direction to simulate a metal backing for the double-layer La_2_Zr_2_O_7_/Eu_2_Zr_2_O_7_ structure, while an open boundary condition was applied at the *z*_max_ direction to provide free-space environment. The incident microwaves (TM mode) were directed perpendicular to the X-O-Y plane. Based on the *R*_L_-*f* curves (Figure 3), the selected monitoring frequencies for *E*-field, *H*-field, and power loss distribution of the double-layer structures are depicted in Figure 5.

RCS, a critical stealth performance metric, quantifies the intensity of radar echoes reflected from a target. It is defined as:

$\mathrm{RCS}\left( \mathrm{dB}m^{2} \right)=10\log(4\pi R^{2}\frac{P_{s}}{P_{i}})$ (11)

where *R*→∞ denotes the far-field distance between the target and the receiving horn antenna, *P*_i_ is the power density of the scattered electromagnetic waves at the receiver, and *P*_r_ is the power density of the incident electromagnetic waves. For RCS simulations, the incident angle (*θ*) was fixed at 90°, while the detection angle (*φ*) varied from -60° to 60° in 2D RCS plots. The size of bottom layer of perfect electric conductor (PEC) was 180 mm×180 mm with a thickness of 1 mm under open boundary conditions. The radar cross section reduction (RCSR) for the double-layer La_2_Zr_2_O_7_/Eu_2_Zr_2_O_7_ structure was determined by subtracting RCS values of the structure from those of the PEC at the corresponding monitoring frequency.


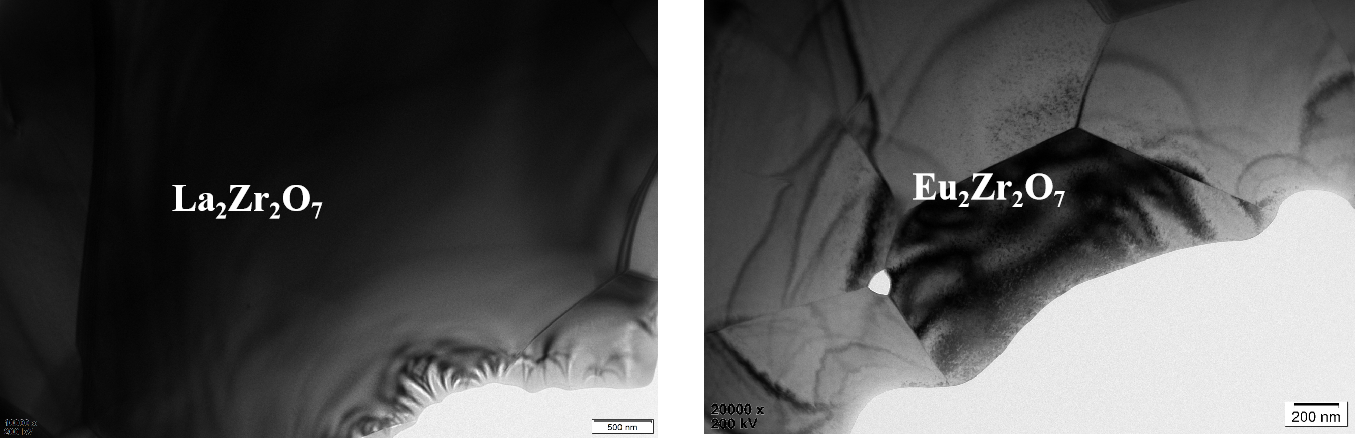


**Figure S1.** Ion-thinning samples for TEM observation


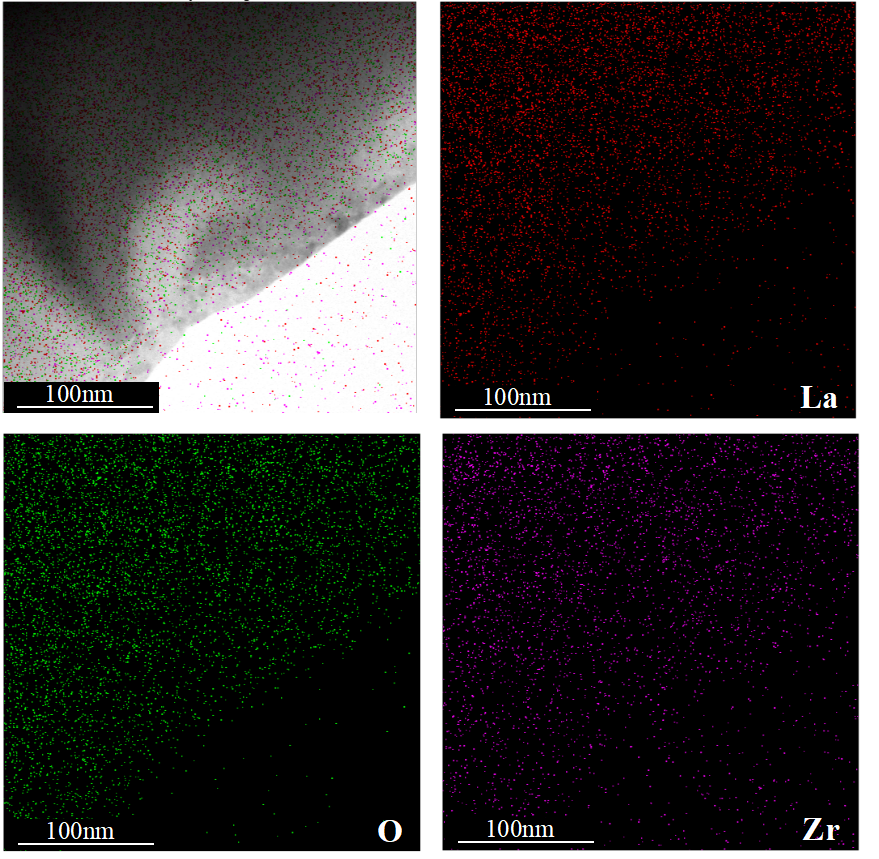


**Figure S2.** TEM-EDS elemental mapping images of La_2_Zr_2_O_7_


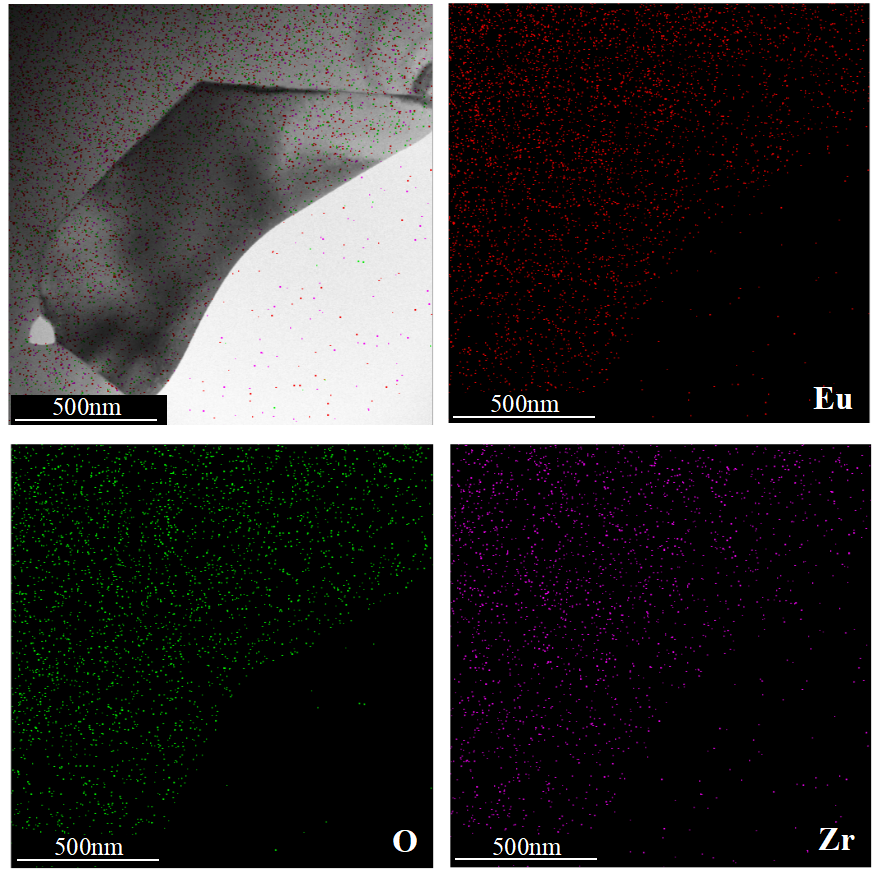


**Figure S3.** TEM-EDS elemental mapping images of Eu_2_Zr_2_O_7_


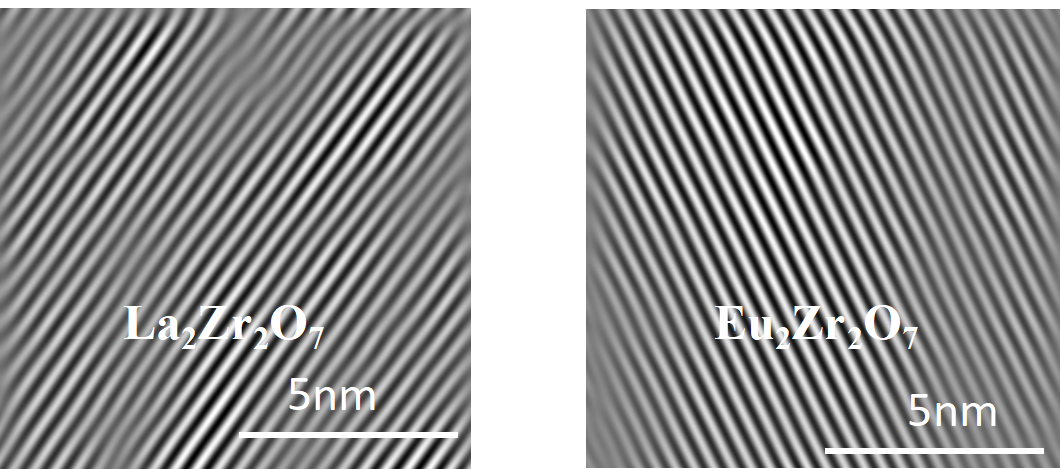


**Figure S4.** The inverse fast Fourier transform (iFFT) filtered image of rare-earth zirconate La_2_Zr_2_O_7_ and Eu_2_Zr_2_O_7_


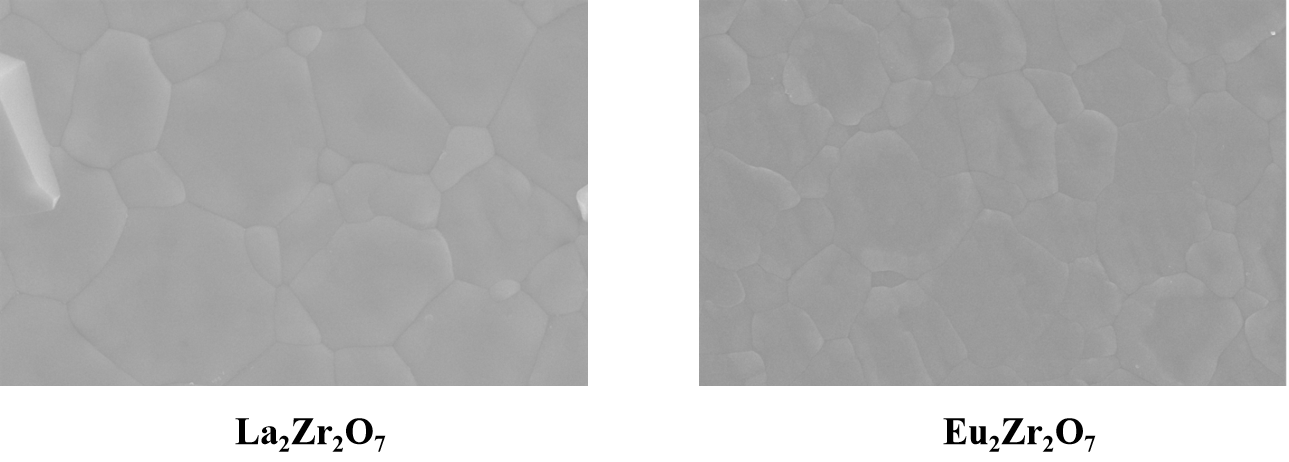


**Figure S5.** SEM images of rare-earth zirconate samples


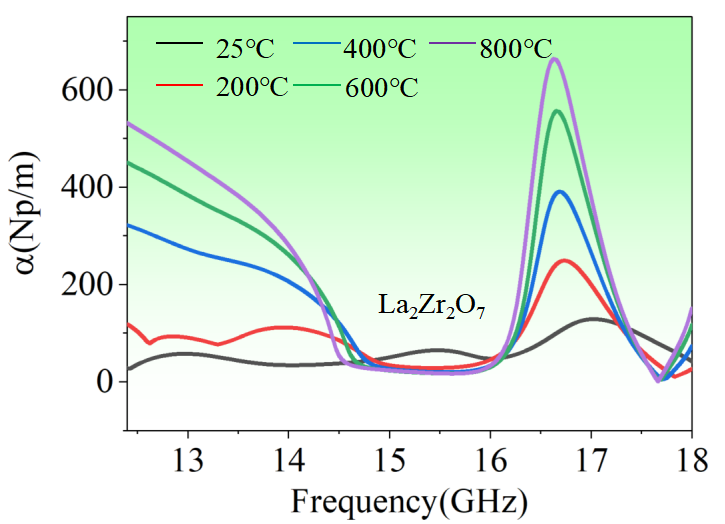


**Figure S6.** Attenuation constant of La_2_Zr_2_O_7_ at RT-800 °C


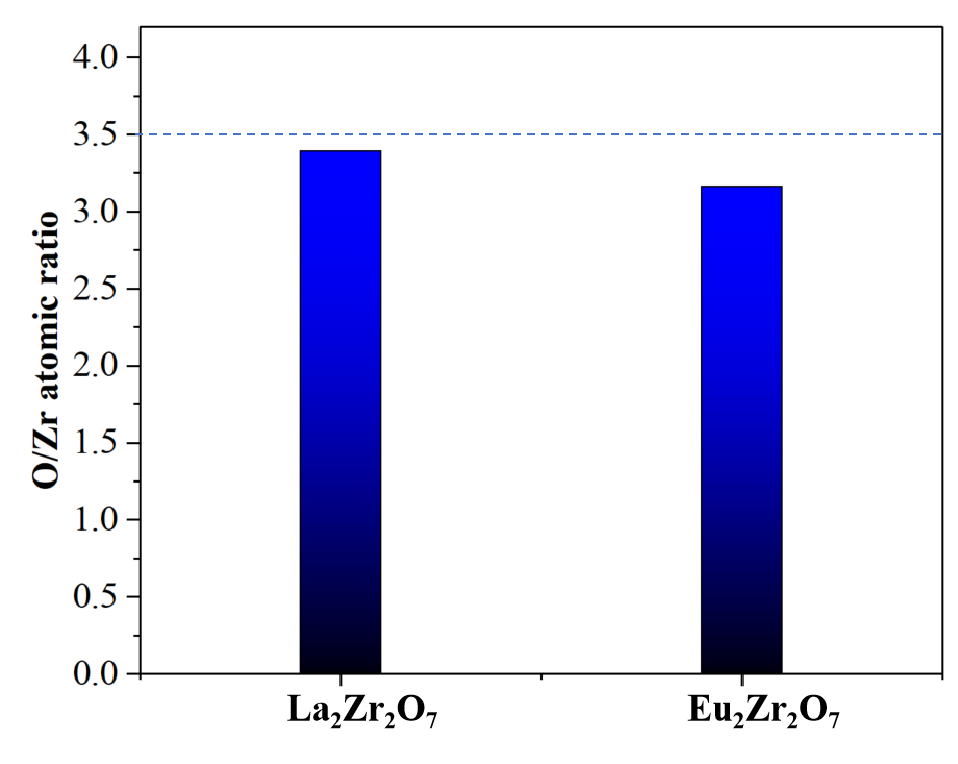


**Figure S7.** Maximum absolute value of valence of oxygen atoms in the La_2_Zr_2_O_7_ and Eu_2_Zr_2_O_7_ unit cells.


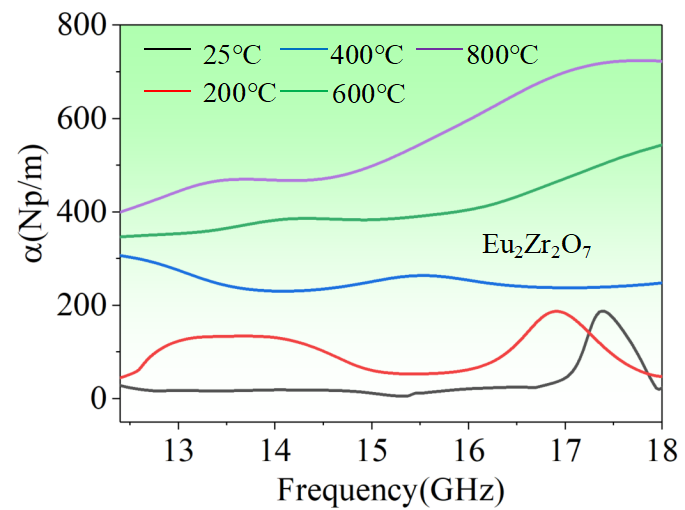


**Figure S8.** Attenuation constant of Eu_2_Zr_2_O_7_ at RT-800 °C


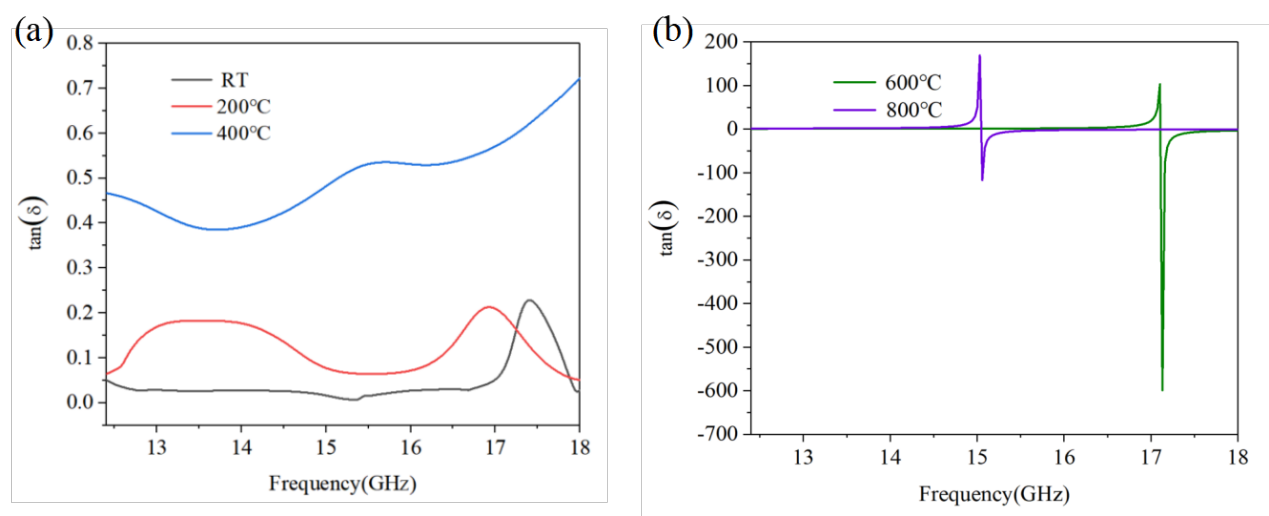


**Figure S9.** Frequency dependence of the dielectric loss tangent of Eu_2_Zr_2_O_7_ across various temperatures


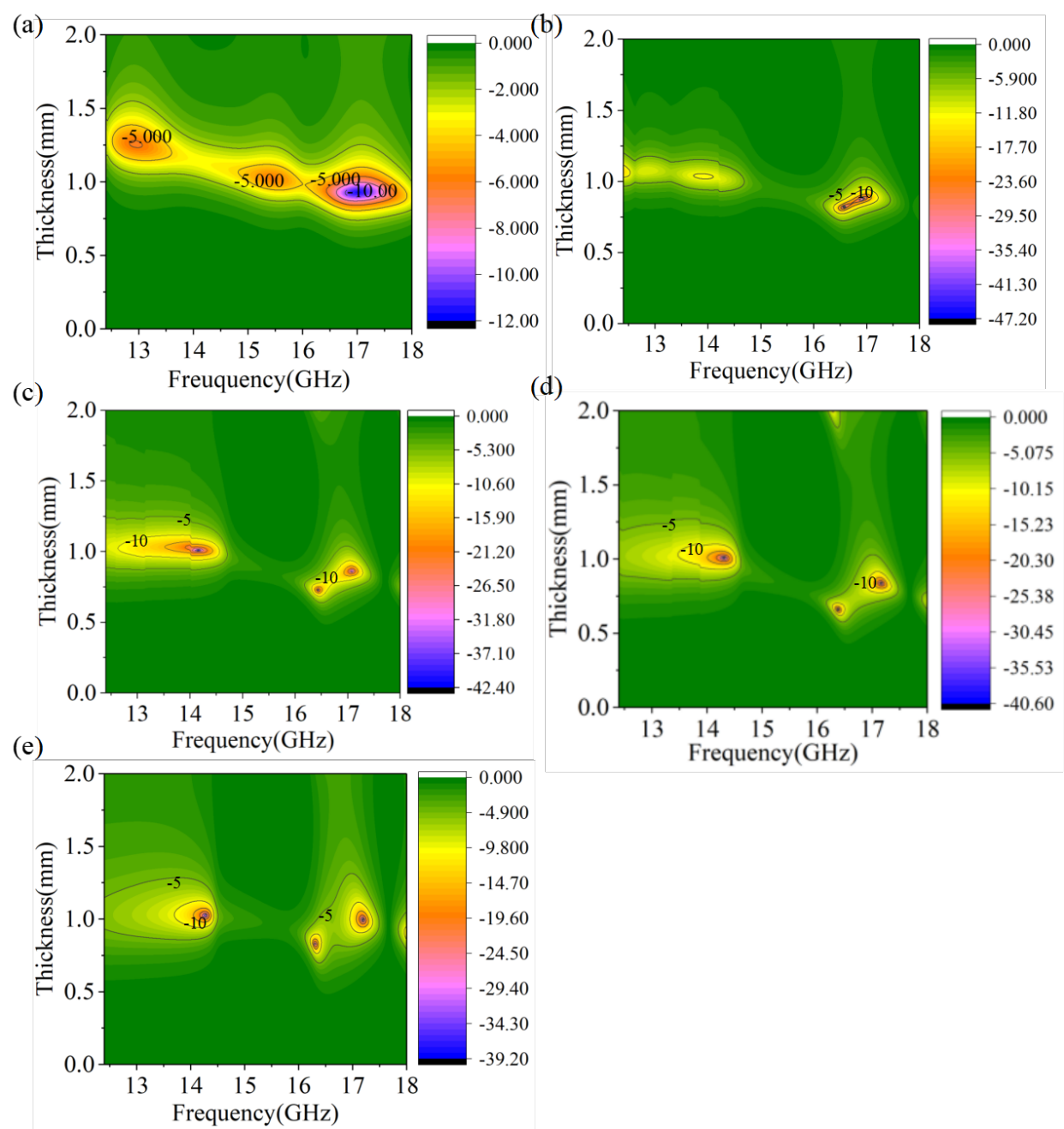


**Figure S10.** Microwave-absorbing 2D plots of single-layer La_2_Zr_2_O_7_ at RT-800 °C in the Ku band.


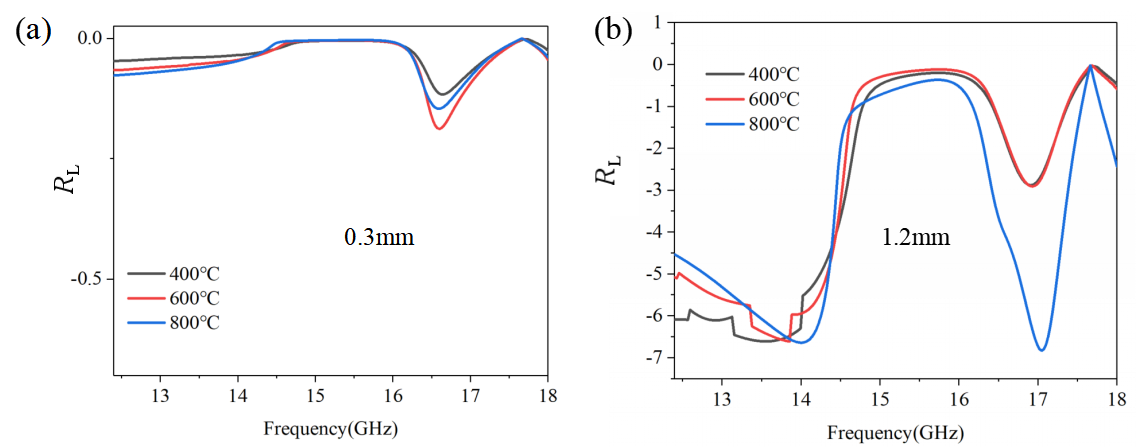


**Figure S11.** The *R*_L_ curves versus frequency and thickness of single-layer La_2_Zr_2_O_7_ with temperature from 400 ℃ to 800 ℃.


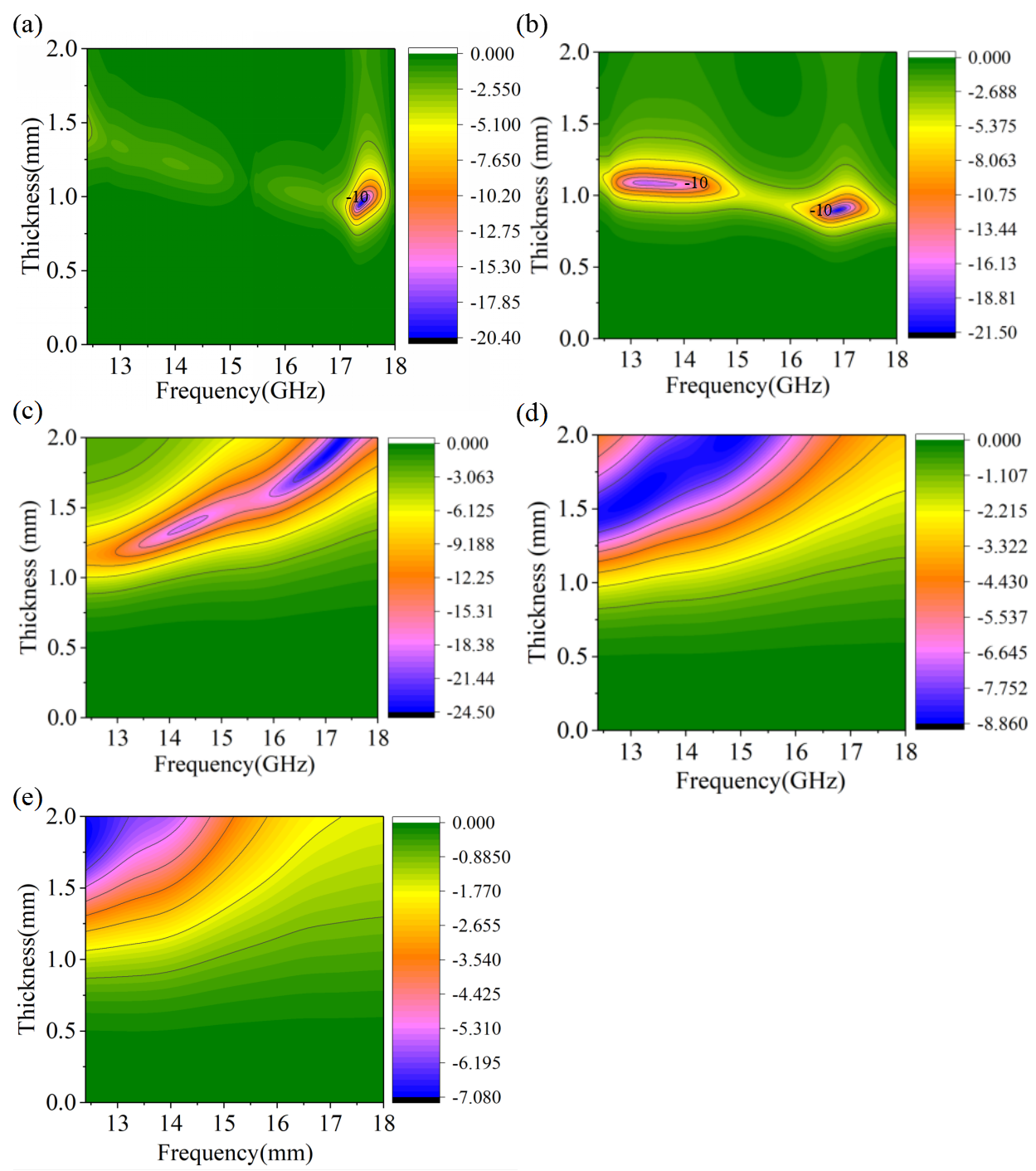


**Figure S12.** Microwave-absorbing 2D plots of single-layer Eu_2_Zr_2_O_7_ at RT-800 °C in the Ku band.

**
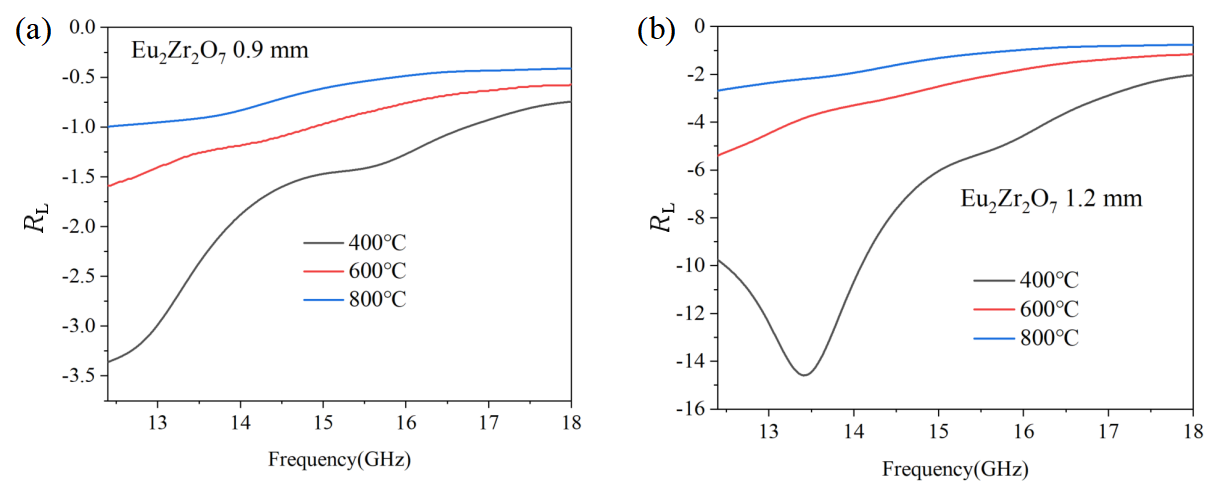
**

**Figure S13.** The *R*_L_ curves versus frequency and thickness of single-layer Eu_2_Zr_2_O_7_ with temperature from 400 ℃ to 800 ℃.


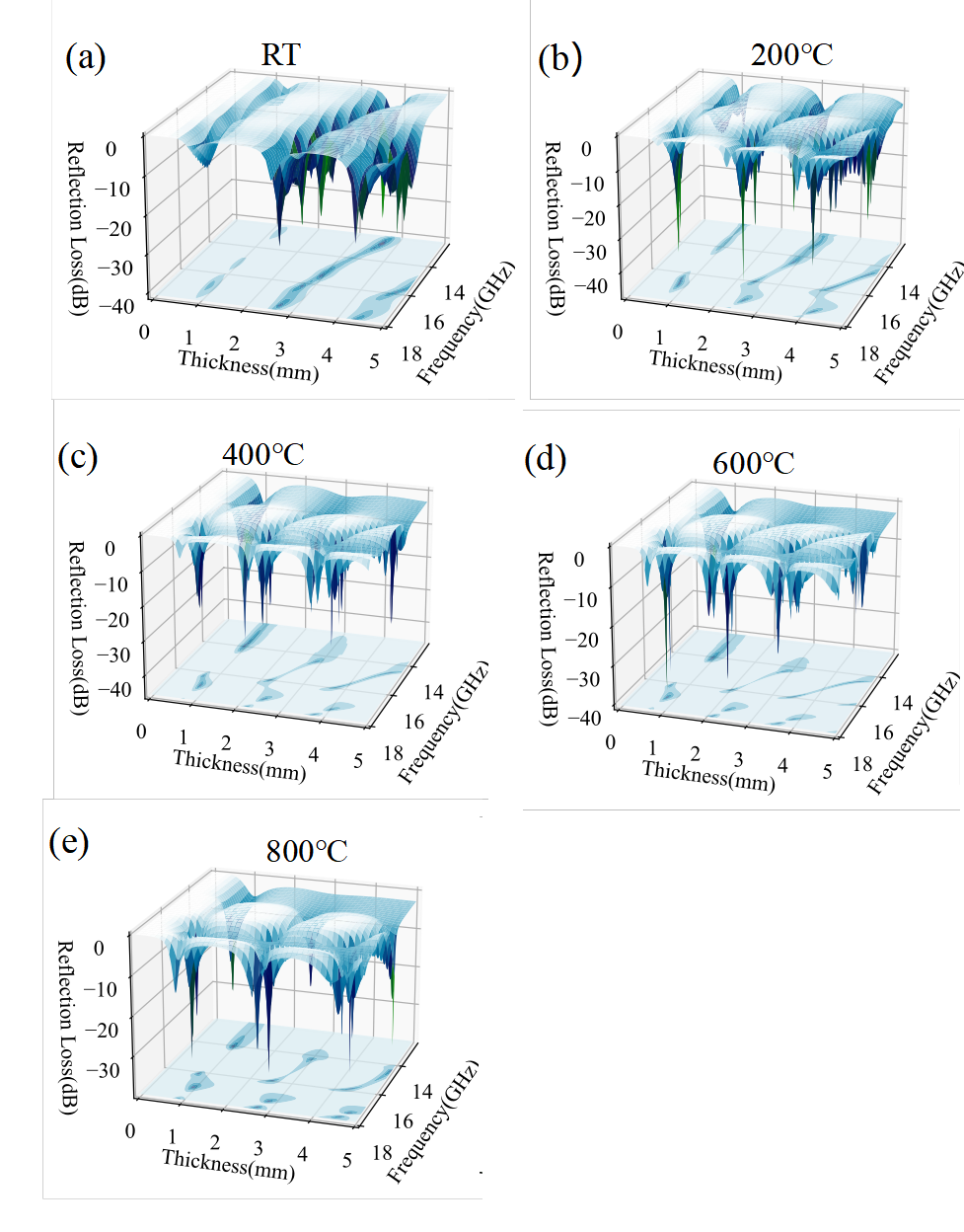


**Figure S14.** 3D *R*_L_ plots of single-layer La_2_Zr_2_O_7._


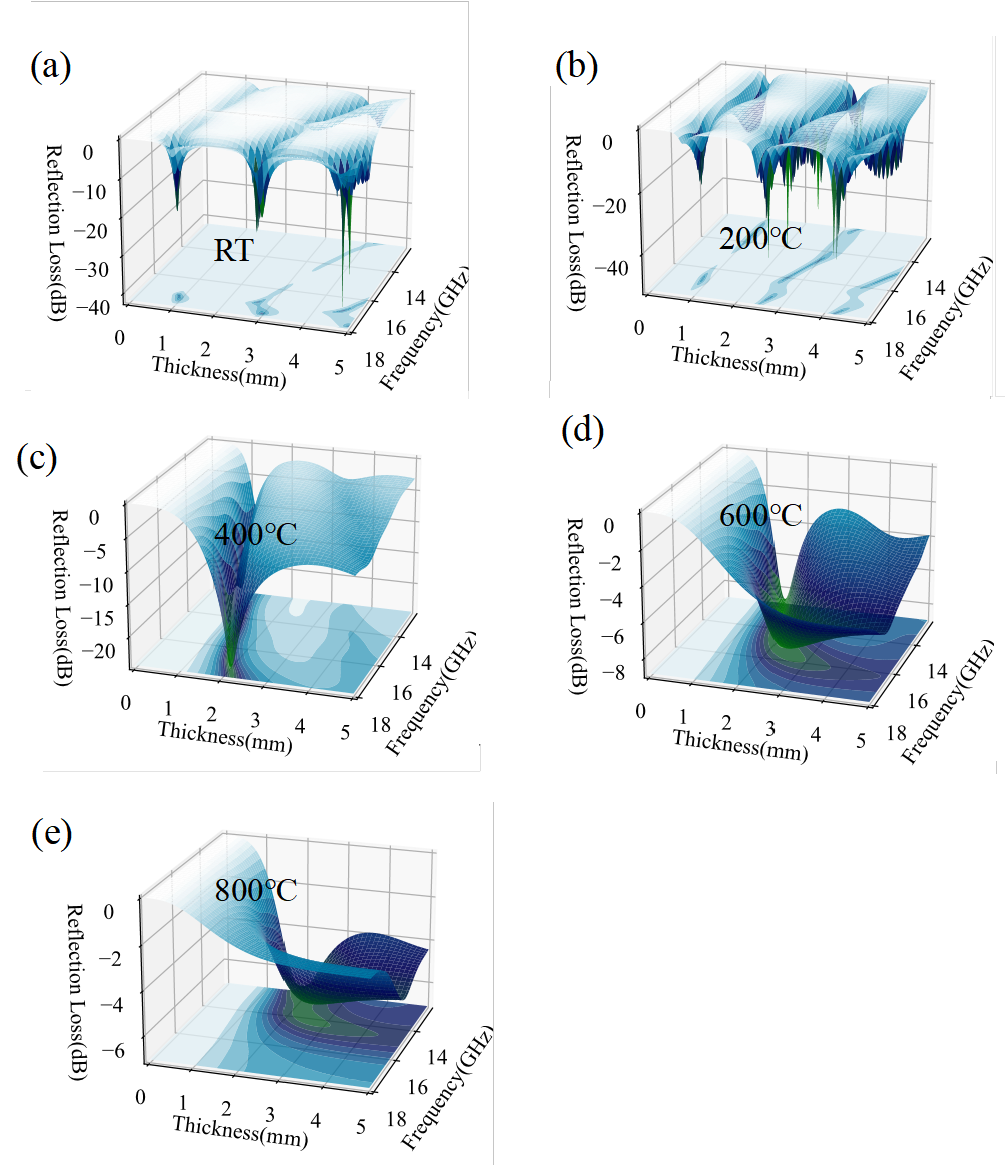


**Figure S15.** 3D *R*_L_ plots of single-layer Eu_2_Zr_2_O_7._


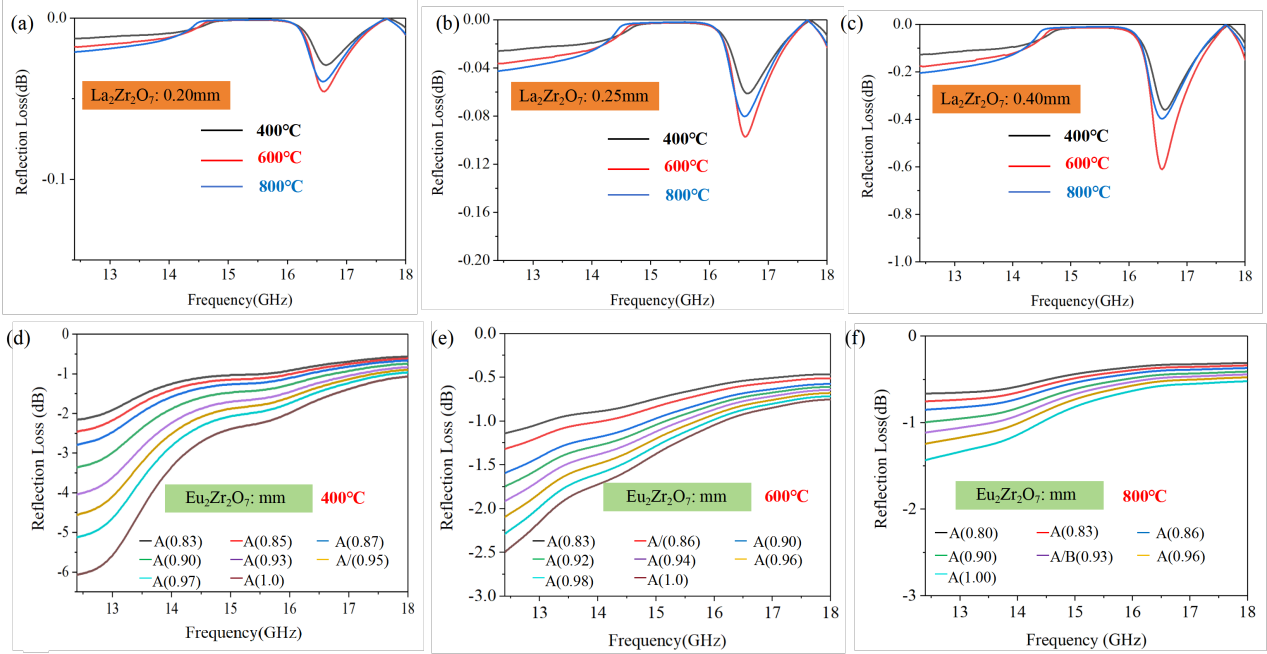


**Figure S16.** Frequency-dependent *R*_L_ curves of single-layer of La_2_Zr_2_O_7_ and Eu_2_Zr_2_O_7_ with different thickness.


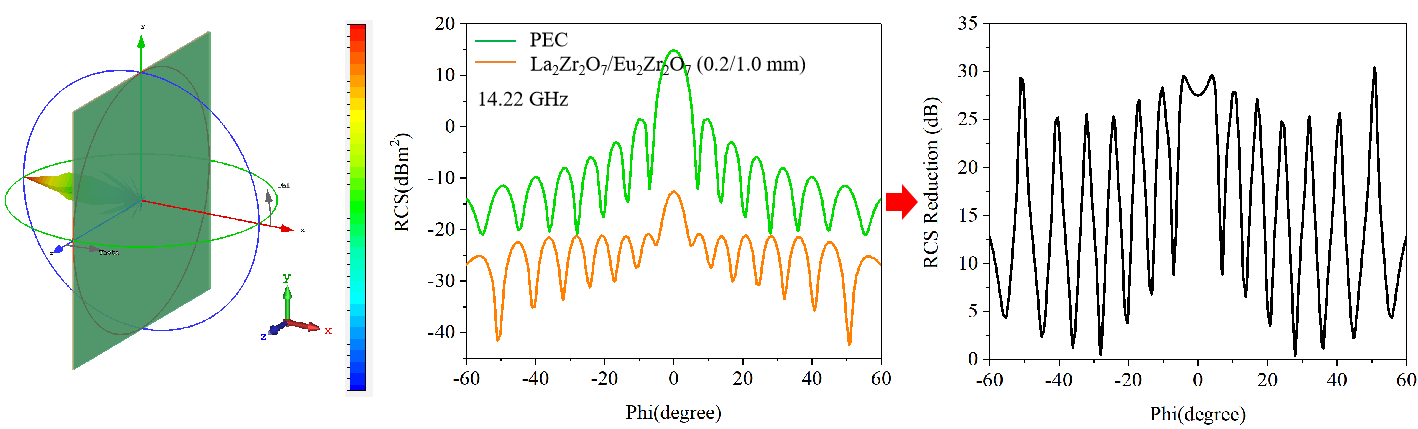


**Figure S17.** 3D RCS images of double-layer structure at 400 °C (0.2 mm/1.0 mm, at 14.22 GHz).

**Table S1.** Ionic conductivity of Eu_2_Zr_2_O_7_ at different temperatures

| Temperature (℃) | *σ*(Eu_2_Zr_2_O_7_)/S·cm^-1^ |
| --- | --- |
| 400 | 5.2615×10^-5^ |
| 500 | 7.5467×10^-5^ |
| 600 | 0.0009555 |
| 700 | 0.004401 |
| 800 | 0.008638 |
| 900 | 0.01427 |
| 1000 | 0.02098 |

**Table S2.** Microwave-absorbing performance (*R*_L_≤-10 dB) of La_2_Zr_2_O_7_ at different temperatures

| Temperature (°C) | Thickness (mm) | Minimum *R*_Lmin_  (dB) | Thickness (mm) | EAB_max_  (GHz) |
| --- | --- | --- | --- | --- |
| RT | 2.72 | -36.93 | 2.72 | 1.56 |
| 200 | 0.82 | -47.10 | 1.06 | 1.17 |
| 400 | 2.26 | -45.75 | 1.02 | 2.06 |
| 600 | 1.01 | -40.48 | 1.02 | 1.06 |
| 800 | 2.57 | -39.66 | 1.00 | 1.09 |

**Table S3.** Microwave-absorbing performance (*R*_L_≤-10 dB) of Eu_2_Zr_2_O_7_ at different temperatures

| Temperature (°C) | Thickness (mm) | Minimum *R*_Lmin_  (dB) | Thickness (mm) | EAB_max_  (GHz) |
| --- | --- | --- | --- | --- |
| RT | 2.73 | -41.99 | 0.96 | 0.39 |
| 200 | 2.69 | -43.67 | 1.08 | 1.81 |
| 400 | 1.95 | -24.47 | 1.53 | 2.51 |
| 600 | 2.08 | -8.90 | - | - |
| 800 | 1.87 | -7.07 | - | - |

**Table S4.** Comparison of microwave-absorbing performance (*R*_L_≤-10 dB) of MAMs reported in recent years

| Temperature  (℃) | MAMs | *R*_Lmin_ (dB) | EAB (GHz) | *d* (mm) | EAB/*d* (GHz/mm) | Ref. |
| --- | --- | --- | --- | --- | --- | --- |
| RT | MXene/Polyimide | 45.08 | 8.58 | 2.1 | 4.08 | 12 |
|  | carbon spring | 36.42 | 9.12 | 2.7 | 3.37 | 13 |
|  | Barium Ferrite | 39.00 | 8.7 | 3.0 | 2.9 | 8 |
|  | fluororubber/carbon | 60.20 | 9.2 | 3.3 | 2.78 | 14 |
|  | Zeolitic Imidazolate | 47.35 | 6.32 | 2.5 | 2.528 | 15 |
| 200 | TiC-Al_2_O_3_/Silica | 49.20 | 1.9 | 1.95 | 0.974 | 16 |
|  | Graphene | 17 | 4.2 | 2.4 | 1.75 | 17 |
|  | CdS-MWCNTs | 47 | 4.2 | 2.6 | 1.61 | 18 |
|  | CNTs/Polyimide | 29.10 | 2.04 | 1.7 | 1.2 | 19 |
| 300 | Multi-walled CNTs | 25 | 2.01 | 1.5 | 1.34 | 19 |
| 400 | CNTs/ZnO | 17 | 3.1 | 2.72 | 1.10 | 20 |
|  | Ti_3_SiC_2_/Al_2_O_3_ | 17.5 | 2 | 2.2 | 0.91 | 21 |
|  | Ti_3_SiC_2_/cordierite | 22 | 1.3 | 1.3 | 1 | 22 |
|  | Ni–SiC | 48 | 4.2 | 2.1 | 2 | 23 |
|  | SiCf/Si_3_N_4_ | 13 | 4.5 | 4.2 | 1.07 | 24 |
|  | Si_3_N_4_–SiC/SiO_2_ | 19 | 4.2 | 3.3 | 1.27 | 25 |
| 500 | Si_3_N_4_–SiC/SiO_2_ | 18 | 4.2 | 3.3 | 1.27 | 25 |
|  | ZnO/ZrSiO_4_ | 17 | 3.1 | 2.86 | 1.08 | 26 |
|  | GO/Si_3_N_4_ | 14 | 4.2 | 4.3 | 0.97 | 27 |
|  | MWCNTs/SiO_2_ | 39.42 | 4.2 | 3.5 | 1.2 | 28 |
|  | LAS/LAS-SiC | 18 | 3.2 | 3 | 1.06 | 29 |
| 600 | Ti_3_SiC_2_/cordierite | 28 | 1.9 | 1.3 | 1.46 | 22 |
|  | SiCf/Si_3_N_4_ | 13 | 4.5 | 4.2 | 1.07 | 24 |
|  | Si_3_N_4_–SiC/SiO_2_ | 17 | 4.2 | 3.3 | 1.27 | 25 |
|  | ZnO/ZrSiO_4_ | 11 | 1.2 | 2.86 | 0.419 | 26 |
|  | TiB_2_/Al_2_O_3_ | 27 | 1.5 | 1.4 | 1.07 | 30 |

**Table S5.** Comparison of microwave-absorbing performance (*R*_L_≤-5 dB) of wide-temperature adaptive MAMs reported in recent years

| Temperature (°C) | MAMs | *d* (mm) | EAB  (GHz) | EAB/*d* (GHz/mm) | *ε*' | Ref. |
| --- | --- | --- | --- | --- | --- | --- |
| 100-400 | Al_2_O_3_/Ti_3_SiC_2_ | 2.2 | 3.9 | 1.77 | 15.4-20.5 | 21 |
| 200-700 | Ti_3_SiC_2_/cordierite | 1.5 | 1.9 | 1.26 | 24.5-37.2 | 22 |
| 100-400 | Ni/ SiC | 2.1 | 4.2 | 2.0 | 18.3-21.9 | 23 |
| 100-600 | Si_3_N_4_(C)/SiC | 2.45 | 4.2 | 1.7 | 10.2-11.9 | 31 |
| 200-600 | SiC_f_/SiC-SiC_nw_ | 2.9 | 4.2 | 1.45 | 6.2-8.2 | 32 |
| 100-500 | Fe-SiC/SiO_2_ | 3.0 | 4.2 | 1.4 | 6.6-8.0 | 33 |
| 200-400 | SiC/SiO_2_ | 4.5 | 4.2 | 0.93 | 5.5-7.2 | 34 |
| 200-600 | SiC_nw_/C/Si_3_N_4_ | 2.9 | 4.2 | 1.45 | 7.5-9.2 | 35 |
| 100-400 | Ti_3_SiC_2_/NASICON | 1.8 | 2.4 | 1.33 | 14-22 | 36 |
| 200-800 | Al_2_O_3_ /TiC | 1.6 | 2.5 | 1.56 | 17.2-28.3 | 37 |
| 200-700 | SiC_f_/SiC/Mo | 2.6 | 3.3 | 1.27 | 6.9-9.7 | 38 |
| 400-700 | Ti_3_SiC_2_/Al_2_O_3_/TiO_2_ | 2.0 | 1.7 | 0.85 | 14.2-21.1 | 39 |
| 400-800 | La_2_Zr_2_O_7_/Eu_2_Zr_2_O_7_ | 1.2 | 4.3 | 3.58 | 30.9-32.3 | This work |

**Supplemental References**

1. A. Fluri, D. Pergolesi, V. Roddatis, A. Wokaun, T. Lippert, In situ stress observation in oxide films and how tensile stress influences oxygen ion conduction. *Nat. Commun.* 2016, 10692.
2. H. Yamamura, H. Nishino, K. Kakinuma, K. Nomura, Electrical conductivity anomaly around fluorite-pyrochlore phase boundary. *Solid State Ionics.* 2003, 158, 359-365.
3. M. Y. Peng, [F. Qin](https://www.gupiaoq.com/citations?user=g__-77oAAAAJ&hl=zh-CN&oi=sra), Dielectric resonance of composites containing randomly distributed ZrB_2_ particles with continuous dual-peak microwave absorption. *Appl. Phys. Lett.* 2024, 124, 262903.
4. H. F. Li, L. Zheng, D. D. Deng, X. L. Yi, X. H. Zhang, X. Luo, Y. B. Wu, W. J. Luo, M. J. Zhang, Multiple natural resonances broaden microwave absorption bandwidth of substituted M-type hexaferrites. *J. Alloy. Compd.* 2021, 862, 158638.
5. Y. Liao, X. Zhou, G. H. He, Quantum dots with Mott-Schottky effect embedded in crystal-amorphous carbon for broadband electromagnetic wave absorption. *J. Alloy. Compd.* 2022, 929, 167246.
6. F. Zhang, N. Li, J. F. Shi, Y. Y. Wang, [D. X. Yan](https://www.gupiaoq.com/citations?user=MeXkp_UAAAAJ&hl=zh-CN&oi=sra), Z. M. Li, Cation Bimetallic MOF Anchored Carbon Fiber for Highly Efficient Microwave Absorption, *Small.* 2024, 20, 2312135.
7. X. Ye, C. Yang, E. Y. He, P. Yang, Q. Gao, T. M. Yan, S. H. Yin, Y. S. Ye, H. H. Wu, Electromagnetic wave absorption properties of the FeSiAl/PLA and FeSiAl-MoS_2_-Graphene/PLA double-layer absorber formed by fused deposition modeling. *J. Magn. Mater.* 2023, 565, 170280.
8. N. Li, Z. Zong, F. Zhang, J. F. Shi, Z. Y. Li, H. K. Xu, Y. F. Zhang, Y. M. Chen, Z. M. Li, Barium Ferrite with High Anisotropy for Ultra-Broadband Microwave Absorption. *Adv. Funct. Mater*. 2025, 35. 2414694.
9. G. Kresse, J. Furthmüller, Efficient iterative schemes for ab initio total-energy calculations using a plane-wave basis set. *Phys. Rev. B.* 1996, 54, 11169-11186.
10. P. E. Blochl, Projector Augmented-wave method. *Phys. Rev. B.* 1994, 50, 17953-17979.
11. G. Kresse, D. Joubert, From ultrasoft pseudopotentials to the projector augmented-wave method. *Phys. Rev. B.* 1999, 59, 1758-1775.
12. X. Wang, X. M. Chen, Q. Y. He, Y. Z. Hui, C. F. Xu, B. C. Wang, F. H. Shan, J. Zhang, J. Y. Shao, Bidirectional, Multilayer MXene/Polyimide Aerogels for Ultra-Broadband Microwave Absorption. *Adv. Mater.* 2024, 36, 2401733.
13. Z. Y. Wang, Z. C. Li, B. Li, A. F. Shi, L. Zhang, Y. B. Zhu, F. Ye, S. H. Yu, Functional Carbon Springs Enabled Dynamic Tunable Microwave Absorption and Thermal Insulation. *Adv. Mater.* 2024, 36, 2412605.
14. C. Yu, B. L. Xie, X. H. Yao, N. Hu, J. H. Guo, X. H. Jiang, A. T. Smith, L. Y. Sun, Cabbage-like flexible fluororubber/carbon aerogel hybrids with negative Poisson’s ratios and excellent microwave absorption. *Matter.* 2023, 6, 4321-4338.
15. Z. Gao, A. Iqbal, T. Hassan, S. Hui, H. Wu, C. M. Koo, Tailoring Built-In Electric Field in a Self-Assembled Zeolitic Imidazolate Framework/MXene Nanocomposites for Microwave Absorption. *Adv. Mater.* 2024, 36, 2311411.
16. Y. Wang, F. Luo, W. C. Zhou, D. M. Zhu, Dielectric and Microwave Absorption Properties of TiC-Al_2_O_3_/Silica Coatings at High Temperature. *J. Electron. Mater.* 2017, 46, 5225-5231.
17. W. Q. Cao, X. X. Wang, J. Yuan, W. Z. Wang, M. S. Cao, Temperature dependent microwave absorption of ultrathin graphene composites. *J. Mater. Chem. A.* 2015, 3, 10017-10022.
18. M. M. Lu, X. X. Wang, W. Q. Cao, J. Yuan, M. S. Cao, Carbon nanotube-CdS core–shell nanowires with tunable and high-efficiency microwave absorption at elevated temperature. *Nanotechnology.* 2016, 27, 065702.
19. H. Y. Wang, D. M. Zhu, W. C. Zhou, F. Luo, High temperature electromagnetic and microwave absorbing properties of polyimide/multi-walled carbon nanotubes nanocomposites. *Chem. Phys. Lett.* 2015, 633, 223-228.
20. L. Kong, X. Yin, M. Han, L. Zhang, L. Cheng Carbon nanotubes modified with ZnO nanoparticles: High-efficiency electromagnetic wave absorption at high-temperatures. *Ceram. Int.* 2015, 41, 4906-4915.
21. Y. Liu, F Luo, J. B. Su, W. C. Zhou, D. M. Zhu, Mechanical, Dielectric, and Microwave-Absorption Properties of Alumina Ceramic Containing Dispersed Ti_3_SiC_2_. *J. Electron. Mater.* 2015, 44, 867-873.
22. J. B. Su, W. C. Zhou, Y. Liu, Y. C. Qing, F. Luo, D. M. Zhu, High-temperature dielectric and microwave absorption property of plasma sprayed Ti_3_SiC_2_/cordierite coatings. *J Mater Sci: Mater Electron.* 2016, 27, 2460–2466.
23. J. Yuan, H. J. Yang, Z. L. Hou, W. L. Song, H. Xu, Y. Q. Kang, X. Y. Fang, M. S. Cao, Ni-decorated SiC powders: enhanced high-temperature dielectric properties and microwave absorption performance. *Powder. Technol.* 2013, 237, 309-313.
24. Q. Zhou, X. W. Yin, F. Ye, Z. M. Tang, R. Mo, L. F. Cheng, High temperature electromagnetic wave absorption properties of SiCf/Si_3_N_4_ composite induced by different SiC fibers. *Ceram. Int.* 2019, 45, 6514-6522.
25. M. Li, X. W. Yin, G. P. Zheng, M. Chen, M. J. Tao, L. F. Cheng, L. T. Zhang, High temperature dielectric and microwave absorption properties of Si_3_N_4_–SiC/SiO_2_ composite ceramics. *J. Mater. Sci.* 2015, 50, 1478-1487.
26. L. Kong, X. W. Yin, Q. Li, F. Ye, Y. Liu, G. Y. Duo, X. W. Yuan, High-temperature electromagnetic wave absorption properties of ZnO/ZrSiO_4_ composite ceramics. *J. Am. Ceram. Soc.* 2013, 96, 2211-2217.
27. Z. X. Zhou, H. Mei, D. Y. Han, L. F. Cheng, Reduced graphene oxide/Silicon nitride composite for cooperative electromagnetic absorption in wide temperature spectrum with excellent thermal stability. *ACS. Appl. Mater. Inter.* 2019, 11, 5364-5372.
28. B. Wen, M. S. Cao, Z. L. Hou, W. L. Song, L. Zhang, M. M. Lu, H. B. Jin, X. Y. Fang, W. Z. Wang, J. Yuan, Temperature dependent microwave attenuation behavior for carbon-nanotube/silica composites. *Carbon* 2013, 65, 124-139.
29. C. H. Peng, P. S. Chen, C. C. Chang, High-temperature microwave bilayer absorber based on lithium aluminum silicate/lithium aluminum silicate–SiC composite. *Ceram. Int.* 2014, 40, 47-55.
30. S. X. Zhao, H. Ma, T. Q. Shao, J. Wang, L. Zhang, S. Sui, M. D. Feng, J. F. Wang, S. B. Qu. Thermally stable ultra-thin and refractory microwave absorbing coating. *Ceram. Int.* 2021, 47, 17337-17344.
31. Z. X. Hou, J. M. Xue, Y. Q. Liu, F. Yang, S. W. Fan, X. M. Fan, L. F. Cheng, Bidirectional periodic pore structure Si-C-N multiphase ceramic with high thermostability and excellent microwave absorption properties over a wide temperature range. *J. Eur. Ceram. Soc.* 2024, 44, 850–857.
32. T. Han, R. Luo, G. Cui, L. Wang, Effect of SiC nanowires on the high-temperature microwave absorption properties of SiC_f_/SiC composites. *J. Eur. Ceram. Soc.* 2019, 39, 1743–1756.
33. X. Y. Yuan, L. F. Cheng, Y. J. Zhang, S. W. Guo, L. T. Zhang, Fe-doped SiC/SiO_2_ composites with ordered inter-ﬁlled structure for effective high-temperature microwave attenuation. *Mater. Design.* 2016, 92, 563-570.
34. X. Yuan, L. F. Cheng, S. W. Guo, L. T. Zhang, High-temperature microwave absorbing properties of ordered mesoporous inter-ﬁlled SiC/SiO_2_ composites. *Ceram. Int.* 2017, 43, 282-288.
35. S. S. Xiao, H. Mei, D. Han, L. Cheng, Sandwich-like SiCnw/C/Si_3_N_4_ porous layered composite for full X-band electromagnetic wave absorption at elevated temperature. *Composites Part B-Eng.* 2020, 183, 107629.
36. D. Chen, F, Luo, W, C. Zhou, D, M. Zhu, Effect of Temperature on Microwave-Absorption Property of Plasma-Sprayed Ti_3_SiC_2_/NASICON Coating. *J. Electron. Mater.* 2019, 48, 1506-1510.
37. T. Shao, H. Ma, J. Wang, M. Feng, M. Yan, J. Wang, Z. Yang, Q. Zhou, H. Luo, S. Qu, High temperature absorbing coatings with excellent performance combined Al_2_O_3_ and TiC material. *J. Eur. Ceram. Soc.* 2020, 40, 2013-2019.
38. H. Gao, F. Luo, Effect of SiC interphase on the mechanical, high-temperature dielectric and high-temperature microwave absorption properties of the SiC_f_/SiC/Mu composites. *Ceram. Int.* 2022, 48, 18567-18578.
39. W. C. Wang, L. Y. Wang, G. Liu, C. Q. Ge, L. Wang, B. Wang, J. Huang, Temperature-dependent dielectric properties and high-temperaturemicrowave absorption performance of Ti_3_SiC_2_/Al_2_O_3_-13%TiO_2_ coatings, *J. Eur. Ceram. Soc.* 2024, 44, 254-260.
